# PANoptosis in the pathogenesis of myelodysplastic syndromes

**DOI:** 10.1002/1878-0261.70309

**Published:** 2026-07-24

**Authors:** Rohit Thalla, Jyoti Lamichhane, Cameron Lewis, Peter Breslin, Jiwang Zhang

**Affiliations:** ^1^ Oncology Institute, Cardinal Bernardin Cancer Center Loyola University Chicago Medical Center Maywood IL USA; ^2^ Department of Cancer Biology Loyola University Chicago Medical Center Maywood IL USA; ^3^ Departments of Biology and Molecular/Cellular Physiology Loyola University Chicago Maywood IL USA

**Keywords:** caspase 8, ineffective hematopoiesis, PANoptosis, Ripk1, Tak1

## Abstract

Myelodysplastic syndromes (MDS) are a heterogeneous group of pre‐leukemic diseases marked by ineffective bone marrow (BM) hematopoiesis, peripheral cytopenia, morphologic dysplasia, and an increased risk of leukemic transformation. Increased programmed cell death (PCD) of hematopoietic stem/progenitor cells (HSPCs) and its associated inflammatory BM microenvironment have been speculated to be one of the major causes of ineffective hematopoiesis. PANoptosis is a collective term for three types of PCD: pyroptosis, apoptosis, and necroptosis. All three are mediated by a very large protein complex called a PANoptosome, composed of the key mediators of the three types of PCD. We reported that the diseased cells in MDS with genetic abnormalities, especially spliceosome mutations, show aberrant hypersensitivity to PANoptotic stimuli. Our study suggests that increased PANoptosis of BM HSPCs is one of the reasons for the ineffective hematopoiesis in MDS patients, and targeting PANoptosis may be a novel treatment strategy for MDS. Here we summarize recent advances in research into PANoptosis and discuss the potential role of PANoptosis in the pathogenesis of MDS. We discuss the potential mechanisms for targeting PANoptotic pathways to treat MDS.

## Graphical abstract

PANoptosis, a combination of three types of programmed cell death, is mediated by a large protein complex called a PANoptosome. In healthy bone marrow hematopoietic cells, PANoptosis is restricted by inhibitory signaling. In MDS, bone marrow cells become sensitive to the PANoptotic stimuli due to the aberrant inactivation of inhibitory signaling or abnormal stimulation of activating signaling. This diagram was created by BioRender.

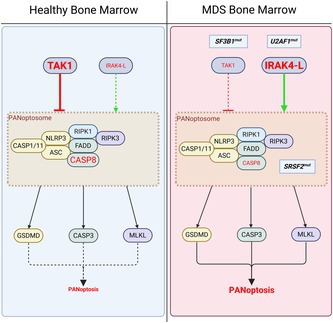

**Abbreviations**
ADAR1: adenosine deaminase acting on RNA 1
AIF: apoptosis‐inducing factor
AIM2: absent in melanoma 2
ALRs: AIM2‐like receptor
ASC: apoptosis‐associated speck‐like protein containing CARD
cFLIP: cellular FLICE (FADD‐like IL‐1β‐converting enzyme) inhibitory proteins
cIAP1/2: cellular inhibitor of apoptosis proteins
CLRs: C‐type lectin receptors
DAMPs: damage‐associated molecular patterns
dsRNA: double‐stranded RNA
EZH2: Enhancer of zeste homolog 2
FADD: Fas‐associated death domain
GSDMD: gasdermin D
IFNs: interferons
IRAK1: IL‐1 receptor‐associated kinase 1
IRF1: interferon regulatory factor 1
LPS: lipopolysaccharide
MDP: muramyl dipeptide
MIF: macrophage migration inhibitory factor
MLKL: mixed lineage kinase domain like
NAIP‐NLRC4: NLR family of apoptosis inhibitory proteins‐NLR family caspase recruitment domain‐containing protein 4
NAIPs: NLR family of apoptosis inhibitory proteins
NEK‐7: NIMA‐related kinase 7
NLR: nucleotide‐binding domain and leucine‐rich‐repeat‐containing
NLRC4: NLR family CARD domain‐containing 4
NLRP1: NLR family pyrin domain‐containing 1
NLRP1, NLRP3, NLRP7, and NLRC4: nucleotide‐binding domain of leucine‐rich repeat (NLR) proteins
NLRs: NOD‐like receptors
NOD2: nucleotide‐binding oligomerization domain containing 2
PAMPs: pathogen‐associated molecular patterns
PCD: programmed cell death
PRG: PANoptosis‐related gene signatures
PRRs: pattern‐recognition receptors
RIG‐I: retinoic acid‐inducible gene I
RIPK3: receptor‐interacting serine–threonine kinase 3
RLRs: retinoic acid‐inducible gene I (RIG‐I)‐like receptors
SARS: severe acute respiratory syndrome
SIRS: systemic inflammatory response syndrome
T3SS ligands: type‐3 secretion system components
T3SS: type 3 secretion system
Tak1: TGF‐β‐activated kinase 1
TLRs: toll‐like receptors
TNF complex IIa: CASP8, RIPK1, FADD
TNF complex IIb: CASP8, RIPK1, RIPK3, FADD
TNFα: tumor necrosis factor α
vMIA: viral mitochondria‐localized inhibitor of apoptosis
xIAP: X‐linked inhibitor of apoptosis proteases
Zbp1: Z‐DNA binding protein 1

## 1 Introduction

Myelodysplastic syndromes (MDS) are a heterogeneous group of preleukemic hematopoietic diseases originating from genetically mutant clonal hematopoietic stem cells (HSCs). The incidence rate of MDS is 4 cases per 100 000 people annually, with a median age at diagnosis of ~ 70 years [1]. Ineffective bone marrow (BM) hematopoiesis, persistent peripheral blood (PB) cytopenia, morphologic dysplasia of blood cells, and a high risk of transformation to acute myeloid leukemia (AML) are the major characteristics of MDS [2].

The primary clinical issues MDS patients face are cytopenias and AML transformation. Most patients suffer from anemia‐related symptoms such as fatigue, dizziness, diminished cardiopulmonary function (shortness of breath, loss of energy, and tachycardia), an increased tendency to cardiopulmonary function failure, and cognitive decline [3, 4]. Many patients suffer from neutropenia and neutrophil dysfunction‐related infections during the course of their disease [5, 6, 7, 8]. A proportion of patients suffer from thrombocytopenia‐related bleeding complications [5]. Approximately 40% of patients die of complications from cytopenia. Transformation to AML occurs in 30–40% of MDS patients, many of whom die within 4–6 months after AML develops [9, 10]. Although the optimal goal of MDS treatment is a cure by means of the complete elimination of mutant clones, due to the lack of medications to specifically target the mutant HSPCs without affecting normal HSPCs, the current goals of MDS treatment are reducing cytopenia‐related symptoms and minimizing morbidity associated with anemia, thrombocytopenia, or neutropenia in order to improve quality of life and extend lifespan [11]. For patients at high risk for AML transformation (HR‐MDS), the treatment goals are to delay AML transformation, prolong survival, and improve quality of life through promoting improvement of PB cell counts [12, 13]. The standard of care for patients with severe anemia is erythropoiesis‐stimulating agents (such as recombinant humanized erythropoietin and darbepoetin‐α) [14, 15] and erythroid differentiation inducers such as luspatercept [16, 17]. For high‐risk patients, the standard of care is to give hypomethylating agents such as intravenous or subcutaneous azacitidine, decitabine, or oral decitabine–cedazuridine [18, 19, 20]. This current standard of care is little more than palliative. Allogeneic HSC transplantation has been approved as, currently, the only potential curative option for the minority of patients with available HSC donors. A better understanding of the disease pathogenesis is critical for designing more effective treatments.

Cytopenia in MDS is a result of ineffective BM hematopoiesis, caused by impaired differentiation/maturation and increased programmed cell death (PCD) of genetically mutant BM HSPCs [21, 22, 23, 24]. In addition, mutant hematopoietic cells (HCs) also produce inflammatory cytokines, creating an inflammatory BM microenvironment which inhibits hematopoiesis among the remaining healthy HSPCs. Clonal evolution and an accumulation of additional mutations in the mutant HSPCs drive a growth advantage in the inflammatory BM environment, which leads to transformation to AML [25]. Increased extrinsic apoptosis, pyroptosis, and necroptosis of BM HCs in MDS have been reported independently by many early studies as an explanation for the ineffective hematopoiesis observed. All three types of PCD can be induced by innate immune signaling and/or inflammatory cytokines, which are highly dynamic and are regulated by intertwined signaling pathways [26]. Recently, we reported that all three types of PCD can be detected in MDS patient BM samples, suggesting PANoptosis [27, 28]. Our studies suggest that targeting PANoptotic pathways represents a potentially effective therapeutic strategy for *SF3B1*‐mutant (*SF3B1* ^*mut*^) MDS [28]. Here, we review the most recent advances in the study of the signaling pathways and master regulators of PANoptosis and introduce an understanding of the potential contributions of PANoptosis to the pathogenesis of MDS. We also discuss potential strategies to target the PANoptotic pathways for what we believe will be improved treatments for MDS. In addition, we discuss the current limitations of PANoptosis research and indicate the potential solutions to be achieved in future studies.

## 2 The concept of PANoptosis

### 2.1 Innate immune responses induce PANoptosome formation and PANoptosis

PANoptosis is a collective term referring to the three types of PCD: pyroptosis, apoptosis, and necroptosis [29, 30]. It was first observed in infectious diseases and was speculated to be stimulated by pathogen‐associated molecular patterns (PAMPs), damage‐associated molecular patterns (DAMPs), and/or inflammatory cytokines such as tumor necrosis factor α (TNFα) and interferons (IFNs) [31, 32, 33, 34]. PAMPs are molecular structures released from pathogens that include lipids, proteins, and nucleic acids, such as lipopolysaccharide (LPS), lipoteichoic acid, viral RNA, and bacterial DNA. DAMPs are endogenous ‘danger molecules’ that include HMGB1, monosodium urate, S100A8/A9, and heme. The molecules that recognize PAMPs and DAMPs in tissue cells are called pattern‐recognition receptors (PRRs), which normally activate innate immunity and inflammation to control the invading pathogens and regulate tissue repair and regeneration [35]. Several families of PRRs have been identified, including Toll‐like receptors (TLRs), retinoic acid‐inducible gene I (RIG‐I)‐like receptors (RLRs), cytosolic DNA sensors (CDSs), C‐type lectin receptors (CLRs), nucleotide‐binding domain and leucine‐rich repeat‐containing receptors (NLRs), and absent in melanoma‐2 (AIM2)‐like receptors (ALRs) [36, 37]. TLRs and CLRs are cell membrane‐associated receptors, while CDSs, RLRs, NLRs, and ALRs are cytosolic nucleotide/toxin sensors. Different types of PRRs recognize distinct types of PAMPs and DAMPs to activate downstream innate immune and inflammatory signaling pathways [38].

**Fig. 1:**
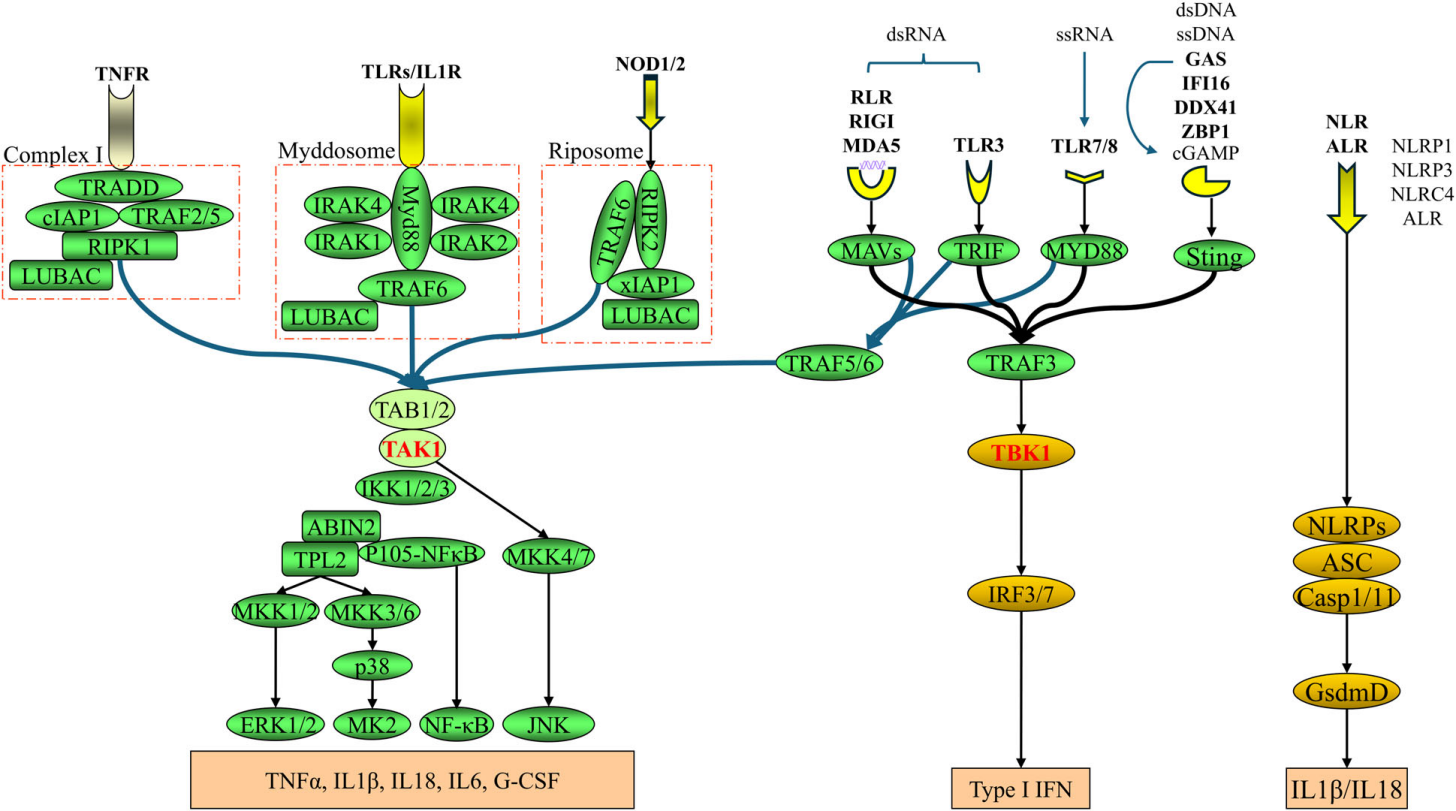
Innate immune signaling pathways. Different pattern‐recognition receptors (PRRs) recognize distinct types of PAMPs/DAMPs to preferentially activate the three signaling pathways for cytokine production. ALR, AIM2‐like receptor; CDSs, cytosolic DNA sensors; IL1R, IL1 receptor; MDA5, melanoma differentiation‐associated protein 5; NLRs, NOD‐like receptors; RLRs, RIG‐I‐like receptors; TLRs, Toll‐like receptors; TNFR, TNF receptor.

**Fig. 2:**
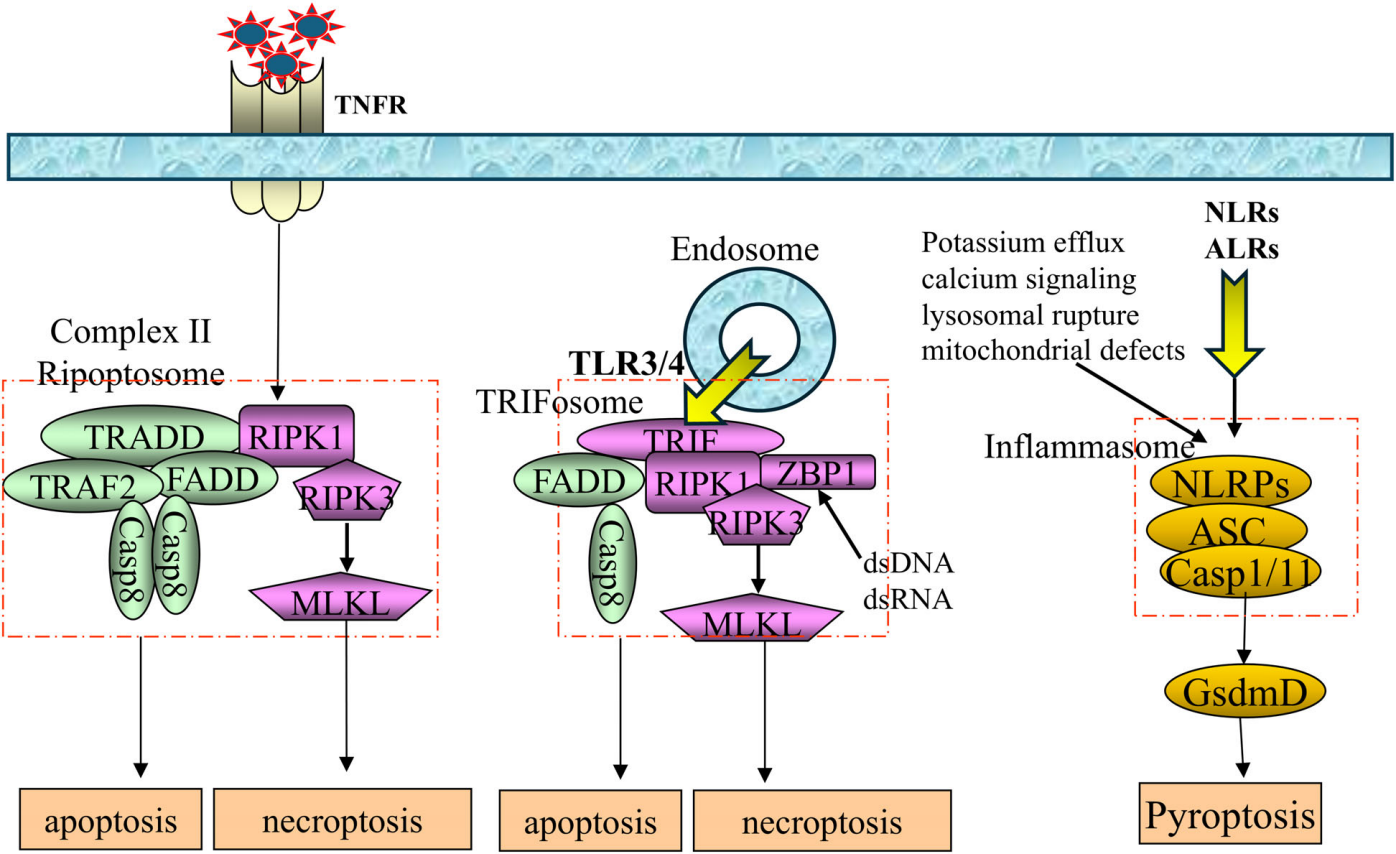
Innate immune response activates programmed cell death (PCD) signaling. Death receptors and TLR3/4 activate extrinsic apoptotic and necroptotic PCD by triggering complex II formation. ZBP1, a cytosolic sensor of both dsDNA and dsRNA, can also activate apoptotic and necroptotic PCD by directly interacting with RIPK1 and/or RIPK3. NLRs and ALRs preferentially activate pyroptosis. Death receptors and TLR3/4 also activate pyroptosis through secondary signaling pathways such as potassium efflux, calcium signaling, lysosomal rupture, and mitochondrial defects.

During infections, to protect themselves against invading pathogens, host cells recognize pathogen‐released PAMPs via their PRRs (cell membrane or cytosolic) to stimulate an immune response by inducing the production of inflammatory cytokines [39]. Three major signaling pathways, TAK1 (MAP3K7), TBK1/IKKε and the inflammasome, have been found to mediate the production of inflammatory cytokines (Fig. 1). TAK1 signaling regulates the expression of many inflammatory cytokines including TNFα, IL1β, IL6, CCL2, CXCL2, and G‐CSF by activating downstream IKKα/β/NEMO‐NFκB, MKK3/6‐p38‐MK2, MKK4/7‐JNK, and MKK1/2‐ERK1/2 signaling pathways [39]. TBK1/IKKε mediates the production of type I and II interferons by activating IRF3/7 signaling [39]. The inflammasome regulates the maturation and secretion of cytokines, including IL1β and IL18 [39]. In addition, the activation of PRRs also induces diverse forms of PCD to eliminate infected host cells [40]. Induced activation of different types of PRRs preferentially induce one or two of these pathways. For example, most TLRs activate myddosome‐mediated TAK1 signaling [41, 42]; TLR3/TLR4 as well as most RLRs and CDSs/ALRs activate TBK1 signaling mediated by TRIF, MAVs, and STING, respectively, while inflammasome‐forming NLRs and some ALRs activate ASC/CASP1/11 (Casp1/4/5 in mice)‐mediated inflammasome signaling and pyroptosis. TLR3/TLR4 also induces TRIFosome‐mediated CASP8‐apoptotic signaling [43] (Fig. 2). Thus, inflammasome‐mediated pyroptosis and/or the CASP8‐mediated extrinsic apoptotic pathway are regularly observed in infected tissues [38], while RIPK1/RIPK3‐MLKL‐mediated necroptosis is only detected in cells with reduced CASP8 activity [44]. PCD contributes to disease pathogenesis during infections by causing tissue damage and releasing DAMPs. DAMPs then bind distinct PRRs to trigger inflammatory cytokine storms, organ damage, and lethality [45, 46]. In many types of pathogen infections (such as in *HSV1, M. tuberculosis, K. pneumoniae, Cryptococcus, Aspergillus, C. albicans, A. fumigatus, C. neoformans*, or *F. novicida* infection), PCD destroys cells infected with these pathogens, as well as damaged cells, in order to prevent the spread of the infection and restore tissue homeostasis by promoting pathogen clearance and anti‐pathogen immune responses [47, 48, 49, 50], whereas in some other pathogen infections (such as β‐CoV, *Y. pestis, M. marinum, L. major, L. monocytogenes*, or *C. neoformans* infection), PCD might facilitate pathogen spread and exacerbate the diseases by inducing positive feedback between cell death and cytokine release and by inducing the death of immune effector cells and evading host defenses [49, 51]. However, PANoptosis only occurs in certain infectious diseases because in normal tissue cells, PANoptosis is restricted by many inhibitory signals such as TAK1 and TBK1. Most infectious diseases which activate PANoptosis are normally associated with severe systemic inflammatory response syndrome (SIRS) in patients [52, 53, 54, 55].

**Fig. 3:**
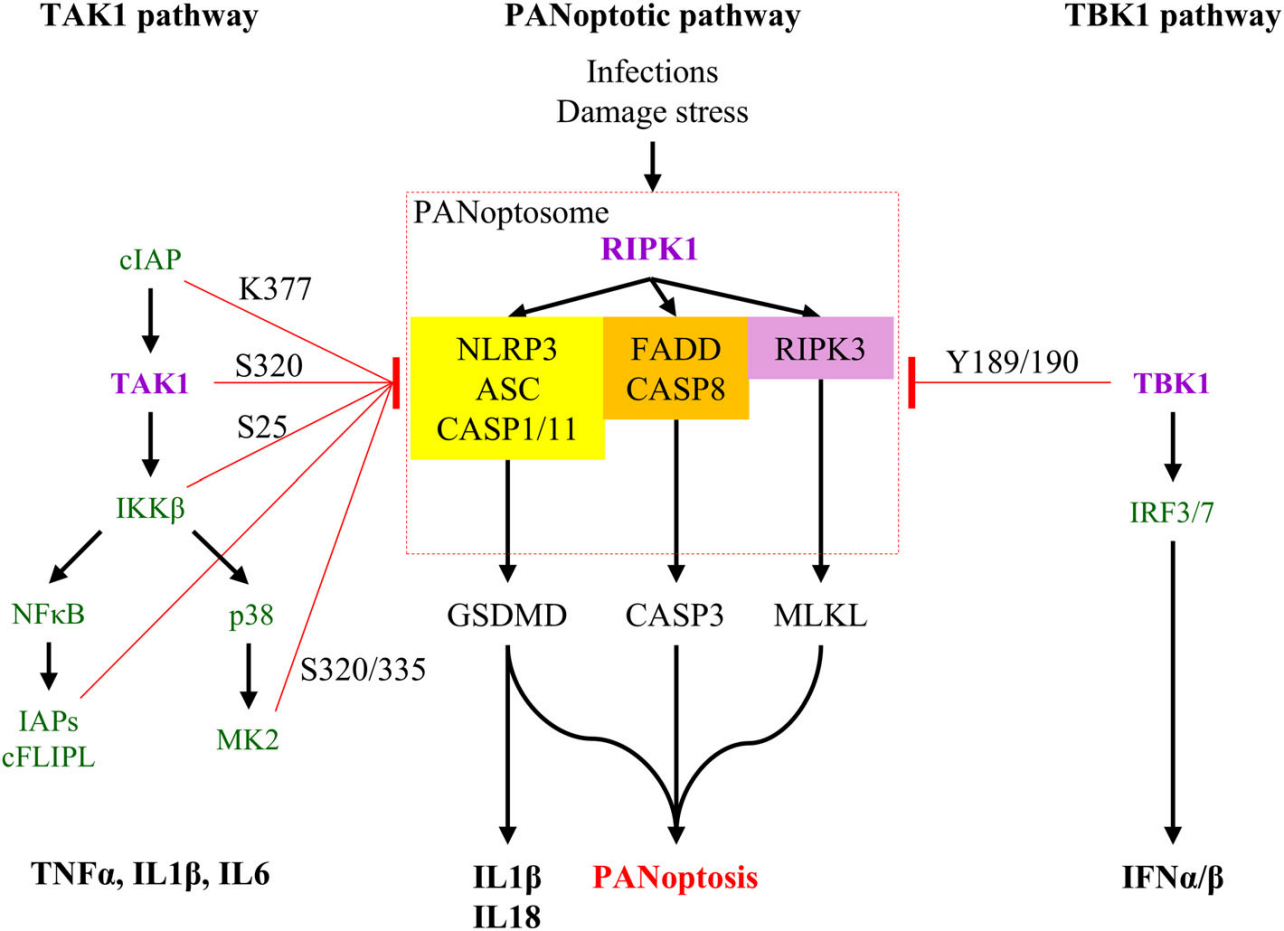
PANoptotic signaling pathways and their negative regulators. Infections or damage stresses induce PANoptosis by triggering the assembly of a PANoptosome. cIAP‐TAK1‐p38/MK2, NFκB signaling, and TBK1 signaling restrict PANoptosis by repressing the activation of RIPK1 or promoting survival gene expression.

PANoptosis has been described in several pathogen infections (Table S1) [56, 57, 58, 59] and has been implicated in the pathogenesis of major human chronic inflammatory diseases including rheumatoid arthritis, inflammatory bowel disease, Aicardi–Goutieres syndrome (AGS) [60], neurological diseases [61, 62], acute lung injury/acute respiratory distress syndrome (ALI/ARDS) [63, 64, 65], psoriasis, and hemolytic diseases [56, 66], as well as cancers [30, 67, 68, 69, 70, 71, 72]. PANoptosis is mediated by a large molecular complex called a PANoptosome, which contains the key signaling components for all three types of PCD (ASC/NLRP3/CASP1/11 for pyroptosis, FADD/pro‐CASP8 for apoptosis, RIPK1/RIPK3 for necroptosis). The PANoptosome provides a type of scaffold platform for the intensive crosstalk among the three PCD pathways (Fig. 3) [33, 34]. GSDMD, CASP3/7, and MLKL are the executors of pyroptosis, apoptosis and necroptosis, respectively, which are activated by upstream mediators. Based on the sensors (PRRs) that trigger PANoptosis, ZBP1, AIM2, NLRP12/NLRC5, and RIPK1, several types of PANoptosomes have been described. ZBP1 uses its Zα1/Zα2 domains to sense cytosolic Z‐RNAs/Z‐DNAs and then recruits RIPK1 and RIPK3 through its RIP homotypic interaction motif (RHIM) to initiate PANoptosome formation [59, 73, 74]; AIM2 uses its HIN domain to sense cytosolic dsDNAs and then recruits ASC through its pyrin domain (PYD) to trigger PANoptosome assembly under facilitation by ZBP1 and Pyrin [75]. Heme, in combination with other PAMPs/DAMPs and/or TNFα via TLR2/4, upregulates NLRP12 and NLRC5 in NAD^+^‐depleting conditions to trigger the activation of the NLRP12/NLRC5 PANoptosome. However, the cytosolic ligands that activate NLRP12 and NLRC5 have not yet been identified [56, 66]. TLR3/4, TNFR, and ZBP1 can function as the sensors for the RIPK1 PANoptosome [33, 34]. Currently, it is unknown whether other PRRs can also function as sensors for PANoptosis. The molecules that are involved in PANoptosome formation contain a PYD (including ASC, NLRP3, AIM2, NLRP12, and Pyrin), a death effector domain (DED, such as pre‐CASP8 and FADD), a RHIM domain (including RIPK1, RIPK3, ZBP1, and TRIF), a caspase recruitment domain (CARD, including ASC, CASP1, NLRC5, and Pyrin), or a death domain (DD, such as FADD and RIPK1) [76]. These proteins build up the PANoptosome through homotypic domain interactions of PYD, CARD, DED, RHIM, and DD. Within all these types of PANoptosomes, CASP8 functions as the ‘switch’ among the three types of PCDs. CASP8 mediates apoptosis and regulates pyroptosis but restricts necroptosis by cleaving RIPK1 and RIPK3 [44]. CASP8 inactivation induces increased levels of RIPK1 and RIPK3 proteins, resulting in hypersensitivity to necroptosis. The role of CASP8 in pyroptosis is cell context‐dependent.

### 2.2 The key regulators of the PANoptotic pathway

RIPK1, TAK1, and IRF1 have been identified as master regulators of PANoptosis [29]. RIPK1 contains both a DD and a RHIM allowing it to integrate into all types of PANoptosomes by directly interacting with FADD, RIPK3, ZBP1, and/or TRIF. In many situations, PANoptosis is dependent on the activation of RIPK1 kinase (RIPK1‐dependent), while in some other situations, PANoptosis is dependent on the scaffolding activity of RIPK1 independent of its kinase activity (RIPK1‐independent).

TAK1 is a member of the MAP3K family, which can be activated by a diverse set of intracellular and extracellular stimuli including TNFα, TGFβ, IL1, TLR ligands, and Nod1/2 ligands. These stimuli, functioning through their corresponding receptors, trigger the assembly of multiple signaling proteins into high‐order oligomeric complexes, collectively known as supramolecular organizing centers (SMOCs), followed by the specific E3 liganse‐mediated addition of K63‐linked polyubiquitination and M1‐linked linear ubiquitination chains to the components of the SMOCs. The K63 and M1‐ubiquitination chains form K63/M1‐Ub hybrids which provide interactional docks to recruit the TAK1/TAB1/2 complex and its downstream substrates including NEMO/IKKα/β and ABIN2/p105‐NFκB/TPL2 complexes for signaling activation. The SMOCs stimulated by TNFα are called TNFR signaling complex I (TRADD‐TRAF2/5‐cIAP1/2‐RIPK1 complex). The SMOCs stimulated by TLRs/lLR are referred to as myddosomes (MYD88‐IRAK1/4‐TRAF6 complex) (Fig. 1) [77, 78, 79], and the SMOCs stimulated by TLR3/4 through TRIF are called TRIFosomes (TRIF‐ZBP1‐RIPK1‐FADD‐CASP8 complex) (Fig. 2). In TNFR‐complex I, TRAF2/5‐cIAP1/2 and LUBAC mediate K63‐linked polyubiquitination of RIPK1 on its K377 residue and M1‐linked linear ubiquitination of RIPK1 on its K627 residue, respectively [80, 81, 82]. In the myddosome and the TRIFosome, both TRAF6 and Pellinos 1/2 contribute to the formation of K63‐linked polyubiquitination chains to IRAK1 and other components of the myddosome and RIPK1 in the TRIFosome, while LUBAC adds M1‐linked polyubiquitin chains.

TAK1 plays essential roles in regulating cell survival and inflammation and is a master regulator of PANoptosis. TAK1 signaling restricts both RIPK1‐dependent and RIPK1‐independent PANoptosis [83, 84]. In addition to regulating the expression of inflammatory cytokines, TAK1 also regulates the expression of survival genes such as *xIAP*, *cIAP1/2*, *BCL2*, *BCL2‐A1*, and *cFLIP* by activating its downstream signaling [85, 86, 87]. Expression of these survival genes inhibits TNFR/TLR‐stimulated RIPK1‐independent PANoptosis. Furthermore, several components of the TAK1 signaling pathway also restrict RIPK1‐dependent PANoptosis [88]. cIAP1, cIAP2, TRAF2/3/5/6, and LUBAC function upstream of TAK1 and restrict RIPK1 activity by mediating K63‐linked ubiquitination of RIPK1 on K377 [80, 89] and M1 linear ubiquitination of RIPK1 on K627 [81, 82]. TAK1, IKKs/NEMO, and MK2 restrict RIPK1 activity by phosphorylating RIPK1 on either S320 (TAK1 and MK2) or S6/25 (IKKs/NEMO) [54, 86, 90, 91, 92, 93, 94]. In murine bone marrow‐derived macrophages (BMDMs), pharmacological or genetic inhibition of Tak1 results in spontaneous Ripk1‐dependent PANoptosis stimulated by autocrine TNFα [55, 95]. The spontaneous PANoptosis in *Tak1*‐deficient cells can be nearly completely prevented by Ripk1 inhibition. However, upon pathogen infection, or LPS or polyI:C induction, *Tak1*‐deficient BMDMs undergo Ripk1‐dependent and Ripk1‐independent PANoptosis which can be partially prevented by Ripk1 inhibition and largely prevented by Ripk1 depletion, suggesting a Ripk1 scaffolding activity‐mediated PANoptosis [96]. Although noncanonical Ripk1 autophosphorylation on its T169 residue drives Casp8‐mediated GsdmD cleavage and pyroptotic cell death in *Tak1*‐deficient cells [97], Casp8/Ripk3 deletion can only partially prevent such cell death, and Casp8/Casp1/11‐Ripk3 deletion is required to fully prevent cell death, suggesting that both Casp8 and Casp1/11 mediate pyroptosis in such cells [34, 53, 54, 55, 95, 96, 98, 99, 100, 101, 102]. Detailed analysis suggests that *Tak1*‐deficient cells are hypersensitive to LPS‐ and polyI:C‐induced TRIFosome formation which triggers PANoptosis by recruiting ASC‐NLRP3‐CASP1/11 to the inflammasome and RIPK3‐MLKL for necroptosis. Previous studies demonstrated that inactivation of cIAP1/2, LUBAC, IKKβ, or MK2 can also cause overactivation of RIPK1. Future study needs to determine whether inhibition of any of these molecules can also trigger PANoptosis.

RIPK1 activity can also be restricted by several other kinase‐mediated types of phosphorylation. For example, TBK1 restricts RIPK1 activation by phosphorylating RIPK1 on its T189/T190 residues [103, 104]. During autophagy and energy stress, ULK1, AMPK, and JAK1/Src restrict death receptor signaling‐induced RIPK1 activity by phosphorylating RIPK1 on residues Ser357, Ser416, and Tyr384, respectively [105, 106]. Whether inactivation of any of these kinases can also push hypersensitive cells toward PAMP/DAMP‐stimulated PANoptosis needs to be determined experimentally. In addition, whether co‐inhibition of any of these inhibitory kinases can synergistically promote PANoptosis is an open question. Although CASP8 is necessary for PAMP‐ and DAMP‐induced extrinsic apoptosis, CASP8 also regulates necroptosis and pyroptosis. Cells deficient in CASP8 express increased levels of RIPK1 protein and are hypersensitive to TNFα, IFNα, IFNγ, polyI:C, and LPS‐induced necroptosis and pyroptosis. These studies suggest that CASP8 limits PANoptosis by restricting necroptosis.

In contrast, the phosphatases that remove the inhibitory phosphorylation groups from RIPK1 as well as the deubiquitinases that remove the K63‐linked and M1‐linked ubiquitin chains from RIPK1 are positive regulators of RIPK1 activity and possibly positive regulators of PANoptosis. For example, the phosphatases PPP1R3G/PP1γ and protein phosphatase 6 (PP6) dephosphorylate RIPK1 on S25/S320/S335 and S320, respectively [107, 108], and the CYLD, OUTULIN and A20 E3 ubiquitin ligases remove polyubiquitin from RIPK1. In addition, splicing regulators PTBP1 and RAVER1 promote the expression of the canonical *RIPK1* transcript via suppressing alternative splicing; thus, they can be considered positive regulators of PANoptosis [109]. Additionally, IRF1 has been described as a master positive regulator of PANoptosis which is required for all types of PANoptotic induction. IRF1 is a well‐known IFN target gene which transcriptionally regulates the expression of several key sensors (ZBP1, AIM2 and NLRP12) and executors (MLKL and GSDMD) of PANoptosis [56, 57, 110]. Interestingly, IRF1 is not necessary for any of the individual types of PCD induced by a death receptor or TLR signaling, suggesting a unique function of IRF1 in PANoptosis. The detailed mechanism behind this process is largely unknown.

### 2.3 Two ‘hits’, priming and activation, are needed for PANoptotic induction

Most recent studies suggested that a primed condition is required for the induction of PANoptosis, explaining why PANoptosis only occurs in certain pathogen infections. By screening PAMPs and DAMPs that can induce PANoptosis in murine BMDMs, Sundaram et al. found that no single, individual PAMP or DAMP was able to induce PANoptosis [56]. PANoptosis can only be induced by certain combinations of two PAMPs, PAMP+DAMP or PAMP+TNFα. In such combinations, one of the PAMPs or DAMPs primes the cells to PANoptosis by inducing the expression of IRF1 and its target genes: *ZBP1*, *TRAF12*, *AIM2*, *GSDMD*, and *MLKL* [56]. PANoptosis can be readily induced by the costimulation of another PAMP, DAMP, or inflammatory cytokine in these primed cells. For example, in heme+PAMP/DAMP‐induced NLRP12 PANoptosis, Pam3, R848, or LPS primes the cells by inducing Myd88‐mediated IRF1 and its targeted gene expression. PANoptosis then can be readily triggered by heme costimulation. Heme might also prime the cells to PANoptosis by inducing mitoROS‐dependent NLRP12 expression and NAD^+^ depletion‐dependent NLRC5 expression, which makes the cells hypersensitive to PANoptosis induced by the addition of polyI:C, Pam3, LPS, or R848 [56, 66]. *ZBP1* is a well‐known gene targeted by IFN. In many types of tissue cells, IFNα/β primes the cells to RNA/DNA virus‐induced ZBP1‐mediated PANoptosis by IFNAR1‐STAT1‐IRF9 signaling‐regulated ZBP1 expression [111, 112, 113]. In many types of tumor cells, IFNγ primes the cells to TNFα‐induced RIPK1‐dependent PANoptosis via the activation of STAT1‐IRF1‐NOS2‐NO signaling [34, 53, 56, 114, 115, 116, 117, 118, 119, 120]. In a rheumatoid arthritis model, C1q stimulation primes synovial macrophages to cADPR‐induced NLRC5/NLRP12‐PANoptosis by triggering the translocation of C1QBP to the mitochondrial matrix to drive metabolic exhaustion and NAD^+^ depletion [121]. Several stress conditions can also prime cells to PANoptotic inductions. For example, heat stress induces the expression of NINJ1, a transmembrane protein that can disrupt cell membranes during cell death processes [122, 123]. Owing to this mechanism, under heat stress conditions (42°C.), microphages are supersensitive to PAMP‐induced PANoptosis [124]. In addition, in the normoxic condition, RIPK1 protein levels are limited by EGLN1–pVHL axis‐mediated ubiquitination‐associated proteasomal degradation [125]. FADD is also limited by ubiquitination‐associated proteasomal degradation [126]. However, hypoxic stress downregulates EGLN1 which results in elevated levels of RIPK1 protein. Hypoxic stress also induces FADD SUMOylation which prevents ubiquitination and degradation of FADD. Thus, a hypoxic environment primes the cells for PAMP‐induced PANoptosis [125, 126]. Under oxidative stress situations, elevated ROS prime cells to PANoptosis by promoting the formation of disulfide bonds between RIPK1 C257, C268, and C586 residues, which enhances RIPK1 S161 autophosphorylation and activation [127].

**Fig. 4:**
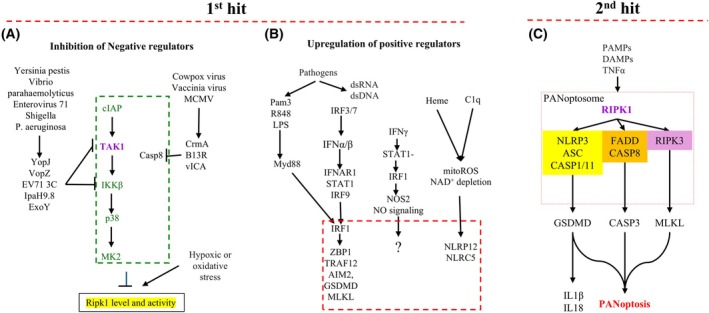
A schematic diagram to demonstrate the ‘two hits theory’ of PANoptosis. Pathogen‐associated molecular patterns (PAMPs) released from pathogens or damage‐associated molecular patterns (DAMPs) released from damaged cells stimulate the assembly of a PANoptosome. Two hits (2 PAMPs, 2 DAMPs, or 1 PAMP+ 1 DAMP) are needed to trigger the dynamic assembly of PANoptosomes in normal cells. (A, B) The first hit primes cells to PANoptosis by inhibiting negative regulators of the PANoptosome such as TAK1, IKKβ, or Casp8, all resulting in elevated Ripk1 protein level or activity (A), or by upregulating positive regulators or mediators of PANoptosomes such as IRF1, ZBP1, AIM2, GSDMD MLKL, NLRP12, and NLRC3 (B). (C) PAMPs, DAMPs, or cytokines, which are the second hits, induce the stepwise assembly of the PANoptosome. As a consequence, more than one PRR can be detected in the PANoptosome.

In many infections, pathogens prime the cells by releasing the molecules that can repress the negative regulators of the PANoptotic pathway. Many pathogens produce molecules that inhibit TAK1 and NEMO/IKKβ, such as YopJ released by *Yersinia pestis*, EV71 3C cysteine protease secreted by *Enterovirus 71* [128], VopZ produced by *Vibrio parahaemolyticus* [129], ExoY secreted by *P. aeruginosa* [130] and IpaH9.8 produced by *Shigella* [131]. Many other pathogens produce CASP8 inhibitory molecules, such as, CrmA, B13R (also called SPI‐2) and vICA (viral inhibitor of Casp‐8 activation) produced by Cowpox virus, Vaccinia virus and MCMV, respectively [132]. In cells with *TAK1* or *CASP8* deficiency, an individual PAMP or inflammatory cytokine is sufficient to induce PANoptosis, suggesting that the priming phase is unnecessary for such cells. Many pathogens also induce the production of types I and III IFN, which stimulate IRF1 expression and cytosolic double‐stranded RNA (dsRNA) and double‐stranded DNA (dsDNA) production, priming cells for PANoptotic induction [32, 113]. In addition, some genetic abnormalities might also prime the cells to stimulate PANoptosis. For example, FUNDC1 is a mitochondrial outer‐membrane protein which plays a critical role in the regulation of mitochondrial integrity. FUNDC1 interacts with TUFM, a key factor in the translational expression and repair of mitochondrial DNA (mtDNA), which stabilizes mtDNA. Cardiomyocytes with FUNDC1 deficiency undergo cytosolic release of mtDNA. In such cells, doxorubicin readily induces PANoptosis without any priming step [133]. Elevated expression of the splicing modulators PTBP1 and RAVER1 also sensitizes cells to PANoptosis by promoting the canonical RIPK1 isoform by repressing alternative isoform splicing [109, 134]. ADAR1 is an RNA editing enzyme which restricts ZBP1‐mediated PANoptosis in tumor tissues [73]. In AGS and ulcerative colitis gut mucosa, cells with *ADAR1* mutations or silencing fail to properly edit dsRNAs, which predispose to MDA5/MAVS‐dependent type I interferon responses [135, 136] and ZBP1‐mediated PANoptosis [135, 137]. The data that support the two hits theory of PANoptosis are summarized in Fig. 4 and Fig. S1.

## 3 The potential role of PANoptosis in MDS pathogenesis

### 3.1 Increased PANoptosis in MDS BM cells

Despite having normal to hypercellular BM, all MDS patients exhibit cytopenia in at least one lineage due to the impaired production of mature blood cells. Most previous studies suggested that this ineffective hematopoiesis is caused by the aberrant activation of innate immune signaling and its associated PCD and inflammatory cytokine production in BM HCs [136, 138, 139, 140, 141]. These studies independently identified increased apoptosis, pyroptosis, and necroptosis in BM HCs to explain ineffective hematopoiesis in MDS patients [138, 142, 143, 144, 145, 146, 147]. Almost all early studies examined PCD in patient BM samples using *in situ* end‐labeling (ISEL) of fragmented DNA, TUNEL staining or cleaved Casp3 staining, suggesting an elevated cell death receptor signal‐mediated extrinsic apoptosis in MDS [142, 148, 149, 150, 151, 152, 153, 154, 155]. The role of pyroptosis and necroptosis in the pathogenesis of MDS was recognized in 2016 by Dr. List's laboratory [145] and in 2019 by Dr. Zinkel's laboratory [147], respectively (Table S2). In a study of BM samples from 25 low to intermediate risk MDS patients, we found that all three types of PCD can be detected in patient BM samples, specifically in patients with mutations of splicing modulators and/or epigenetic modifiers, suggesting an increase in PANoptosis in MDS. BM and PB cells from MDS patients contain an average of 3–4 genetic abnormalities [156]. Mutations of RNA splicing modulators (e.g., *SF3B1*, *SRSF2*, *U2AF1/2*, *ZRSR2*) and epigenetic modifiers (e.g., *TET2*, *DNMT3A*, *IDH1/2*, *ASXL1*, *EZH2*, *BCOR*) are the most common driver mutations in MDS, which occur in > 60% and > 40% of patients, respectively, as detected in patient blood samples [157, 158, 159]. Such mutations might influence the expression and/or function of the components of PANoptotic signaling pathways, providing a primed condition and enhanced sensitivity of the mutant HSPCs to infection‐ or damage‐induced PANoptosis.

### 3.2 PANoptosis in the pathogenesis of SF3B1^mut^ MDS

Heterozygous missense mutation of *SF3B1* has been reported in ~ 20% of all MDS cases, and > 80% of MDS patients with ring sideroblasts (RS) [160, 161]. The mutant SF3B1 molecules in HSPCs primarily affect erythropoiesis, causing refractory anemia with RS [162]. Patients with *SF3B1* mutations are mainly characterized by showing ineffective erythropoiesis with a relatively good prognosis, representing a distinct nosologic entity [160]. Blockage of terminal erythroid differentiation and increased PCD in erythroblasts (EBs) are believed to be the major causes of the ineffective erythropoiesis observed in *SF3B1* ^*mut*^ MDS patients [163, 164, 165, 166]. The terminal differentiation arrest of *SF3B1* ^*mut*^ EBs, as demonstrated by the accumulation of orthochromatophilic EBs together with a severe reduction of reticulocytes in most patients [167] can be partially corrected by treatment with luspatercept, a ligand trap for the TGFβ superfamily [17]. We found increased PANoptosis (with relatively low frequency of necroptosis) in the BM from all 15 *SF3B1* ^*mut*^ MDS patients [28]. Our study suggests that activation of RIPK1‐PANoptosis might be one of the major causes of ineffective erythropoiesis in such disease.

SF3B1 is a subunit of the SF3B protein complex which plays a key role in the recognition and selection of the branch site of pre‐mRNAs in the determination of 3′ splice sites [168]. K700E, K666, and H662 residues in the helical HEAT domains of *SF3B1* are the hotspots that are commonly mutated in MDS [169]. *SF3B1* mutations cause neomorphic functions, resulting in the alternative use of cryptic splice sites of target transcripts. Aberrant splicing of a large number of transcripts has been identified in *SF3B1* ^*mut*^ HSPCs. Among them, the mis‐splicing of *Abcb7*, a key gene for iron homeostasis and *Tmem14c*, a key gene for heme biosynthesis, disrupts iron export from mitochondria to the cytosol and inhibits heme synthesis, which have been determined to be causes of the RS phenotype [170, 171]. Several mechanisms have been proposed to explain the differentiation blockage of *SF3B1* ^*mut*^ EBs, including deactivation of TAK1‐p38MAPK‐MK2 signaling‐associated premature downregulation of GATA1 [172], hyperactive IRAK4‐long isoform‐mediated overactivation of NFκB signaling and inflammatory response [173, 174], altered splicing of the E3 ligase MKRN1‐associated activation of the p53 pathway [175], unscheduled R‐loop formation and replication fork stalling [176], as well as heme deficiency‐related activation of the EIF2AK1‐mediated mitochondrial integrated stress response [167]. We and others recently found that downregulation of TAK1 signaling might be one of the major causes of refractory anemia in *SF3B1* ^*mut*^ MDS as shown by *in vitro* culture of human CD34^+^ HSPCs and an *in vivo* mouse model. The aberrant use of the 3′ splice site for exon 5 of *TAK1* pre‐mRNA has been consistently detected in both human and mouse *SF3B1* ^*mut*^ HSPCs, which results in intron retention and nonsense‐mediated decay of *TAK1* mRNA [172, 173, 177]. Aberrant splicing of 51 − 72% of *TAK1* transcripts was detected in *SF3B1* ^*mut*^ MDS patient BM cells [172].

Using the K562 cell line and normal human CD34^+^ BM cells, Lieu et al. demonstrated that inactivation of TAK1‐p38 MAPK‐MK2 signaling leads to premature downregulation of GATA1 in erythroblasts [172]. The authors proposed that *TAK1* downregulation promotes proteasomal degradation of GATA1 via the MK2/HSP27 pathway [178]. We found that such is the case with *Sf3b1* ^*mut*^ HSPCs. *Tak1* knockdown (*Tak1* ^*KD*^) leads to the overactivation of Ripk1 signaling in mouse HSPCs, which results in spontaneous PANoptosis and premature downregulation of Gata1 in EBs [179]. High GATA1 protein levels are essential for the proliferation and survival of early erythroid precursors, whereas downregulation of GATA1 protein levels is a necessary step for terminal erythroid differentiation [180]. However, the premature downregulation of GATA1 in early EBs induces premature differentiation of erythroid precursors and PCD. In *Tak1* ^*KD*^ cells, overactivation of Ripk1 results in Casp8‐dependent activation of Casp3 and Casp1. Both Casp3 and Casp1 can cleave the Gata1 protein. Mice transplanted with *Tak1* ^*KD*^ HSPCs developed microcytotic anemia, a phenotype that best recapitulates the MDS‐like disease which develops in *Sf3b1* ^*+/K700E*^ mice [28, 181]. Most importantly, Ripk1 inhibition prevents both premature downregulation of Gata1 and PANoptosis in *Tak1* ^*KD*^ EBs in *in vitro* culture. Ripk1 inhibition also resulted in recovered red blood cell counts in the anemia which developed in *Tak1* ^*KD*^ mice. Furthermore, RIPK1 inhibition largely restored colony‐forming ability and normal differentiation to erythroid progenitors from *SF3B1* ^*mut*^ MDS patients. Our study suggests that inhibition of RIPK1 might be an effective way to treat the anemia that is commonly observed in *SF3B1* ^*mut*^ MDS patients [28].

In addition to activating PANoptosis, activated RIPK1 promotes TBK1‐dependent IFNα/β signaling [27, 104, 182] and NF‐κB signaling [183]. Furthermore, activated RIPK1 can also translocate to the nucleus to promote the expression of *NFκB*, *SP1*, and *JUN‐B* target genes by recruiting a RIPK1/BAF complex to enhancers/promotors [184], or to form a mitotic ripoptosome together with CASP8 during mitosis to ensure chromosomal stability by cleaving PLK1 [185]. In most cell types, TAK1 is the master upstream regulator of NFκB signaling; however, in *SF3B1* ^*mut*^ HSPCs, NFκB is activated despite the downregulation of TAK1 signaling [84, 182]. We found that the elevated NFκB signaling in both *SF3B1* ^*mut*^ and *Tak1* ^*KD*^ HSPCs can be reversed by RIPK1 and TBK1 co‐inhibition, suggesting RIPK1‐TBK1‐mediated NFκB activation.

Sustained TGFβ‐SMAD signal activation was detected in many MDS patient samples, as demonstrated by increased p‐SMAD2 [186, 187]. Previous studies suggested that the elevated TGFβ‐SMAD signal in MDS EBs is due to miR‐21‐mediated downregulation of SMAD7 and SKI, the antagonists of TGFβ‐SMAD signaling [188, 189]. TAK1 can be activated by TGFβ family members. Early studies suggested that activated TAK1 inhibits SMAD signaling and fine‐tunes TGFβ signaling activity. We found that the elevated SMAD signaling in *SF3B1* ^*mut*^ and *Tak1* ^*KD*^ HSPCs can also be largely repressed by RIPK1 inhibition. Thus, overactivation of RIPK1 signaling appears to be the cause of most of the molecular alterations observed in *SF3B1* ^*mut*^ HSPCs.

TAK1 is critical for the survival of HSPCs. We have reported that *Tak1* knockout mice (*Tak1* ^*−/−*^) developed acute BM failure and died within 2 weeks of *Tak1* deletion [190]. In addition to PANoptosis [191], a proportion of *Tak1* ^*−/−*^ HSPCs might die of lysosomal‐related causes because the death of *Tak1* ^*−/−*^ HSPCs can only be maximally prevented by inhibition of Ripk3, Casp8, Casp1/11, and cathepsin B. We found that, compared to normal HSPCs, *SF3B1* ^*mut*^ HSPCs from patients are hypersensitive to Smac‐mimetic treatment (inhibiting cIAP‐mediated polyubiquitination) or the inhibition of TAK1 signaling. Our study suggests that Smac‐mimetic treatment or TAK1 signaling inhibition induces synthetic lethality in *SF3B1* ^*mut*^ HSPCs with reduced effects on normal HSPCs, which might provide a safe dosage window to selectively eliminate the *SF3B1* ^*mut*^ disease clones in patients by further promoting the activation of PANoptosis in such mutant cells [28].

### 3.3 PANoptosis in the pathogenesis of SRSF2^mut^ MDS

Heterozygous missense mutations in *SRSF2* are detected in ~ 15% of MDS patients and 30–50% of chronic myelomonocytic leukemia patients. Many patients with *SRSF2* mutations show an MDS/MPN overlap feature. MDS patients with *SRSF2* mutations are associated with increased risk for transformation to AML and predict a worse outcome [162].

SRSF2 is a member of the SR protein family, which is responsible for recognizing the AG dinucleotide at 3′ pre‐mRNA splice sites [168]. SRSF2 regulates exon usage through recognition of exonic splicing enhancers (ESEs). The P95 residue in the linker between the RNA recognition motif and the arginine–serine‐rich domain of SRSF2 is a hotspot that is commonly mutated in MDS patients. Such mutations alter the binding specificity of SRSF2 to ESEs, resulting in the aberrant inclusion or exclusion of exons in transcripts of hundreds of genes [192]. For example, mutant SRSF2 promotes the inclusion of a pseudo‐exon in the *EZH2* transcript [192], the aberrant use of exon7 in the *FYN* transcript [193], the exclusion of exon 6/7 in the *CASP8* transcript [173], the skipping of exon 14 in the *JAK2* transcript [194] and an in‐frame‐preserving cassette exon in the *BCOR* transcript [173, 195]. Animal model studies suggested that the ineffective erythropoiesis which developed in *Srsf2* ^*P95H*^ mice might be induced by low *Ezh2* expression, increased expression of a truncated inactive Jak2 protein or aberrant expression of a FynB isoform in the mutant erythroid progenitors [192, 193, 194]. However, *EZH2* mis‐splicing was not consistently detected by different studies [192, 196, 197], while mis‐splicing of Jak2 was primarily detected in cells with *Jak2* ^*V617F*^ *Srsf2* ^*P95H*^ compound gene mutations [194]. More recently, Jutzi et al. demonstrated that elevated expression of FynB promotes proliferation and blocks differentiation of erythroid progenitors which might contribute to ineffective erythropoiesis observed in *Srsf2* ^*P95H*^ mice. Fyn has a FynT isoform, which possesses stronger kinase activity and a FnyB isoform, which has reduced kinase activity. FynT is an important regulator of EPOR signaling which activity is required for both homeostatic and stress‐induced erythropoiesis [198]. The elevated FynB isoform in *Srsf2* ^*P95H*^ erythroid progenitors activates mTORC1 pathway, causing reduced proliferation signatures and heme metabolism. Importantly, the proliferation and differentiation defects in *Srsf2* ^*P95H*^ erythroid progenitors can be largely prevented by the inhibition of mTOR signaling. In addition, other groups found that increased expression of *S100a8*/*S100a9* and enhanced R‐loops might also contribute to the ineffective erythropoiesis in *Srsf2* mice [199, 200]. *SRSF2* ^*mut*^ MDS is commonly associated with increased PB monocytes, as shown by the MDS/MPN overlap feature. In a study of HSPCs derived from isogenic human iPSC models, Wheeler et al. demonstrated that the *SRSF2* ^*mut*^ form drives the splicing of a hyperactive long isoform of GNAS (GNAS‐L, a member of the heterotrimeric family of G proteins) which activates ERK/MAPK signaling [201]. Xu et al. showed significantly enhanced mis‐splicing of cell cycle and DNA repair transcripts in both human and murine *SRSF2* ^*P95H/+*^ samples which drive CDK6‐dependent proliferation and survival of the mutant cells [202]. It needs to be determined which of these signal activation events induce the myeloid‐biased differentiation of HSPCs and skew granulocyte‐monocyte progenitors toward development to monocytes.

CASP8 downregulation is a common feature of *SRSF2* ^mu*t*^ cells. In low‐grade MDS patients, CASP8 is downregulated in BM HCs, specifically in CD71^+^ erythroid precursors, which is associated with increased RIPK1 protein and RIPK1‐RIPK3 mediated necroptosis [146]. Inhibition of RIPK1 improves erythroid colony‐forming ability in human MDS, while *Ripk1* over‐expression causes inflammatory cell death and an MDS‐like phenotype in mice [146, 147]. These studies suggest an important role for RIPK1‐RIPK3‐MLKL necroptosis in the pathogenesis of MDS in the early stages [146, 147, 203]. We found increased PANoptosis in the BM HCs from all 6 *SRSF2* ^*mut*^ MDS patients, as demonstrated by a high frequency of necroptosis and pyroptosis with a relatively low frequency of apoptosis [28]. Recently, we reported that *Casp8* ^*−/−*^ mice also developed MDS‐like diseases due to increased Ripk1 levels, Ripk3‐necroptosis and Tbk1‐Ifn/NFκB signaling. In *in vitro* culture, both necroptosis and activated Tbk1‐Ifn/NFκB signaling in *Casp8* ^*−/−*^ HSPCs can be largely reversed by Ripk1 inhibition, suggesting a Ripk1‐dependent mechanism to account for ineffective hematopoiesis [27]. The anemic phenotype of *Casp8* ^*−/−*^ mice better recapitulates the phenotype observed in *Srsf2* ^*P95H/+*^ mice [27]. Our study suggests that CASP8 downregulation may contribute to the ineffective hematopoiesis observed in MDS with *SRSF2* mutations. Thus, we speculate that RIPK1 inhibition might attenuate cytopenia‐related symptoms in *SRSF2* ^*mut*^ MDS. However, the role of RIPK1 signaling in the transformation of *SRSF2* ^*mut*^ MDS to AML is unknown. In hematopoietic malignancies, inactivating mutations of *CASP8* (within the P10 subunit) were reported in 58.21% (85/146) of AML cases, and these are correlated with resistance to chemotherapy and poor prognosis for patients [204]. Future study is needed to determine whether RIPK1‐RIPK3 is downregulated during disease progression and whether inactivation of necroptotic signaling contributes to high risk for AML transformation in *SRSF2* ^*mut*^ MDS. Nevertheless, as is the case with *SF3B1* ^mut^ MDS, we found that a Smac mimetic or TAK1 inhibitor treatment induces synthetic lethality to *SRSF2* ^*mut*^ HSPCs (Zhang et al., unpublished work). Therefore, we believe that further stimulating the activation of PANoptotic signaling might be a better strategy to treat *SRSF2* ^*mut*^ MDS by selectively eliminating the mutant clones.

We speculate that elevated RIPK1 levels and inactivation of CASP8 signaling are not limited to *SRSF2* ^*mut*^ MDS [146]. Analyzing gene expression in MDS samples, *RIPK1* mRNA is found to be overexpressed in some cases, particularly in MDS with multilineage dysplasia. High *RIPK1* expression is associated with severe anemia and predicts hypomethylating agent (HMA) responsiveness compared to low RIPK1 expression cases [203]. Increased *RIPK1* mRNA is associated with adverse prognosis and upregulation of genes involved in TNFα/NFκB signaling, immune signaling and IFN production [203]. Furthermore, increased cFLIPL‐to‐cFLIPS/cFLIPR isoform switching was reported in MDS patients [57, 205, 206]. cFLIPL regulates the activity of CASP8 and prevents CASP8‐mediated apoptosis without affecting necroptosis. However, cFLIPS and cFLIPR have strong CASP8 inhibitory capacity as elevated cFLIPS and cFLIPR expression manipulates *CASP8* knockout, which not only prevents CASP8‐mediated apoptosis but also promotes necroptosis. Therefore, targeting PANoptotic signaling might also be beneficial to patients with RIPK1 upregulation and/or elevated cFLIPS and cFLIPR.

### 3.4 PANoptosis might contribute to the pathogenesis of MDS with aberrantly elevated inflammasome and/or TLR‐Myd88 signaling

U2AF1 is a member of the serine/arginine‐rich protein family which governs alternative splicing of transcripts by recognizing the AG splice acceptor dinucleotide at the 3′ end of introns [207]. U2AF1 also has some splicing‐independent noncanonical functions. For example, U2AF1 in the cytosol regulates the translation of distinct sets of target transcripts (*e.g*., *IL8*, *IL1α*, *NPM1* and nuclear‐encoded mitochondrial mRNAs) by binding to translation start codons in the 5′‐UTR of transcripts to repress translation initiation [208, 209, 210] and interacting with the cleavage factor Im (CFIm) complex in the 3′UTR of transcripts (*e.g*., ATG7) to selective use cleavage and polyadenylation sites [211]. Heterozygous missense mutations in *U2AF1* are detected in 11.7% (5–21.7%) of MDS patients [212, 213]. The S34 and Q157 residues in the first and second zinc finger domains of U2AF1 are the two hotspots that are most commonly mutated in MDS patients [207]. Such mutations cause structural changes in U2AF1, altering the specificity of splicing site binding. Because the structural changes caused by these two hot spot mutations are not the same, the profiles of mis‐spliced transcripts in HSPCs with these two *U2AF* mutations only partially overlap [195]. Most studies revealed that MDS patients with *U2AF1* mutations are younger with an age‐dependent trend. MDS patients with *U2AF1* mutations are characterized by PB cytopenias, including anemia, reduced platelet counts, and an increased percentage of myeloblasts in the BM, specifically with an *S34F/Y* mutation [214]. MDS patients with *U2AF1* ^*S34*^ mutations have relatively low platelet levels, whereas hemoglobin concentrations were lower in MDS patients with *U2AF1* ^*Q157/R156*^ mutations, and myelofibrosis was more common. *U2AF1* ^*mut*^ MDS patients are more likely to have low‐risk karyotypes such as an isolated +8 karyotype, or del(20q). The *U2AF1* ^*Q157P*^ mutation has a higher incidence of chromosome 7 abnormalities [157, 159].

Mis‐splicing of CASP8 has been reported in *U2AF1* ^mut^ cells, resulting in a shorter N‐terminal isoform of the protein; however, the biological significance of such mis‐splicing is unclear [173, 195]. Nevertheless, enhanced alternative splicing of *IRAK4* is consistently detected in *U2AF1* ^mut^ cells and it leads to an elevated expression of the IRAK4‐L isoform. Compared to the canonical IRAK4‐S isoform, IRAK4‐L is more potent in forming myddosomal complexes. Consequently, the TLR‐MYD88‐IRAK‐TRAF6‐NFκB signaling pathway is over‐activated in *U2AF1* ^*mut*^ cells [215]. We also found increased PANoptosis in 2 MDS patients with *U2AF1* ^*mut*^ MDS. Although overactivation of TLR‐MYD88 signaling cannot directly activate PANoptosis, it might provide a primed condition for mutant HSPCs toward infection‐ or damage‐induced PANoptosis. *In vitro* studies suggested that in BMDMs, activation of TLR2 or TLR4 induces a Myd88‐dependent upregulation of IRF1 and its downstream target genes (including *ZBP1*, *AIM2*, *NLRP12*, *MLKL*, and *GSDMD*) [56, 57], which primes the cells to a secondary stimulus to trigger the activation of PANoptosis [56]. Nevertheless, the role of PANoptosis in the pathogenesis of *U2AF1* ^*mut*^ MDS still needs to be verified experimentally. In *in vitro* culture, *U2AF1* ^*S34F*^ expression impairs erythroid differentiation and skews granulocyte differentiation of human HSPCs [216]. In a mouse model, *U2af1* ^*S34F*^ expression induces macrocytic anemia and a persistent reduction of white blood cell counts (marked reduction in B cells) due to the skewed differentiation of early progenitors and increased PCD [217]. Increased accumulation of R loop and replication fork stalling [200, 218], elevated FOXO3a‐mediated target gene expression and its associated NLRP3 inflammasome activation [219], increased secretion of inflammatory cytokine such as IL‐8 and IL‐1α [208], as well as mitochondrial dysfunction (due to ATG7 protein downregulation‐related defective autophagy or enhanced translation and localization of nuclear‐encoded mitochondrial mRNA) [211] have been proposed as the potential mechanisms of ineffective hematopoiesis in *U2AF1* ^*mut*^ MDS. It is unknown whether these biological alterations can also contribute to prime the mutant HCs to PANoptosis.

Overactivation of TLR‐Myd88 and innate immune signaling is a common feature among many MDS patients [220, 221, 222]. Increased concentration of DAMPs including S100A8 and HMGB1 have been reported in BM and PB sera of MDS patients [138, 145, 223, 224]. These DAMPs are either passively released by necrotic cells or actively secreted by stromal cells and activated immune cells [145, 223, 224, 225]. Many genetic abnormalities and epigenetic events in MDS cause enhanced sensitivity of mutant HSPCs to TLR ligand stimulation. For example, haploinsufficiency of the intrinsic TRAF6 regulators *miR‐146a* and *TIFAB* in del(5q) MDS leads to overactivation of the TLR‐MYD88‐IRAK‐TRAF6 signaling pathway [215, 226]. Furthermore, IFNγ and TNFα levels are elevated in the sera of most MDS patients [227]. *IRF1* is the best understood target gene in the IFN signaling entity. IFN stimulation primes many types of cancer cells to LPS‐ and TNF‐induced PANoptosis [73, 113, 228]. IRF1 is required for PANoptotic stimulation by almost all types of stimuli [32, 56]. Future study needs to examine the expression of IRF1 and its target genes in different types of MDS patients to determine in which types of MDS are HSPCs primed for PANoptotic stimulation.

## 4 Future prospective

Due to the interconvertibility of the three types of PCD mediated by the PANoptosome, targeting PANoptosis therapeutically has become a very active area of research for both autoimmune/inflammatory diseases and cancers. In autoimmune/inflammatory diseases, researchers want to reduce immune stimulation and inflammatory reactions by inhibiting the inflammatory types of pyroptosis and necroptosis. In cancers, investigators intend to kill apoptosis‐resistant tumor cells by inducing necroptosis or pyroptosis, simultaneously stimulating antitumor immune reactions in the tumor microenvironment. However, PANoptosis may be a double‐edged sword in different cancers and other diseases. In tumor tissues, inducing PANoptosis in these cells not only directly kills the cells but also stimulates antitumor immunity by releasing immunogenic antigens and cytokines/chemokines for recruiting/activating DCs, CD8^+^ cytotoxic T cells, M1 macrophages and natural killer cells, as well as reprogramming immunosuppressive cellular networks [229, 230, 231, 232]. However, the PANoptotic cancer cells might also release factors that induce the recruitment/expansion of immune repressive cells such as Treg cells, myeloid‐derived suppressor cells and M2 macrophages, resulting in immune tolerance to tumor cells, resulting in their evading immune surveillance. In addition, PANoptosis can also impair antitumor immunity if it occurs in immune cells [233]. Gene expression and immune infiltration analysis of patient samples suggest a distinct correlation between PANoptosis‐related gene signatures (PRG) and immune landscape patterns in different types of tumor tissues. In most types of cancer, including thyroid cancer [234], lower grade glioma [235], gastrointestinal malignancies (biliary tract cancer, colorectal carcinoma, hepatocellular carcinoma, and gastric cancer) [236, 237, 238], high‐risk renal clear cell carcinoma [239], and breast cancer [240], elevated PRG is associated with a worse patient prognosis, and is negatively associated with antitumor immune elements (activated DCs, M1 macrophages, resting mast cells, and memory CD4^+^/CD8^+^ T cells) and positively correlated with protumor immune components (M0/M2 macrophages, activated mast cells, and neutrophils), suggesting a protumor activity of PANoptosis. In cancer types such as cutaneous melanoma [235], aggressive glioma [231], clear cell renal cell carcinoma [241], prostate adenocarcinoma [233], and hepatocellular carcinoma [242], elevated PRG is associated with favorable prognosis and is positively associated with antitumor immunity and negatively related to protumor immunity, implicating a tumor repressive activity of PANoptosis. Thus, further detailed studies are needed to determine how to selectively trigger PANoptosis in cancer cells and elucidate how PANoptotic cells reprogram the tumor immune environment.

### 4.1 Inhibition of RIPK1 for MDS treatment

Due to the dysregulation of master regulators of the PANoptotic signaling pathway, increased PANoptosis in HSPCs is a common feature in MDS, specifically patients with spliceosome mutations. Our study suggests that the increased PANoptosis in MDS with spliceosome mutations is primarily RIPK1‐dependent, and it might contribute to ineffective hematopoiesis and cytopenia. Animal studies suggested that Ripk1 activity is not required for development and tissue regeneration [243, 244, 245]. Many RIPK1 inhibitors have been developed and several of them have been tested in clinical trials for the treatment of various inflammatory diseases. Clinical studies have suggested that most RIPK1 inhibitors can effectively function without obvious adverse events [246, 247, 248, 249]. We believe that inhibition of RIPK1 might be a potential avenue by which to resolve cytopenia‐related symptoms in low‐risk *SF3B1* ^*mut*^ MDS patients. Inhibition of RIPK1 not only prevents PCD but also represses other PCD‐independent functions of RIPK1, such as TBK1‐IFNα/β signaling [27, 182] and TBK1‐NFκB signaling [183], as well as nuclear activities of RIPK1 [184, 185] which may also contribute to the cytopenic and dysplastic phenotype of MDS. For example, in the nucleus, RIPK1 regulates cell cycle‐controlled PLK1 degradation. PLK1 is a master regulator of mitosis, playing a critical role in the proper division of early erythroid precursors and enucleation and maturation of later EB stages [250]. Both inhibition and overactivation of PLK1 have been implicated in abnormal chromosome segregation and genomic instability [175, 251].

However, there are two concerns for RIPK1 inhibition in the treatment of MDS patients. First, during infections, PAMPs also induce RIPK1‐independent PANoptosis [52]. In such cases, the effect of RIPK1 inhibition might be limited. Secondly, for high‐risk MDS cases, RIPK1‐mediated PANoptosis might exert a tumor suppressive mechanism which can prevent the transformation of MDS to AML. Thus, in such MDS cases, RIPK1 inhibition might cause disease progression and hasten AML development.

### 4.2 Enhancing PANoptosis to eliminate disease clones for MDS treatment

The optimal goal for MDS treatment is to fully eliminate mutant clones without affecting normal HSPCs. In *Tak1* ^*KD*^ MDS‐like models, we found that PANoptosis is primarily detected in lineage‐committed progenitors and erythroblasts, while the diseased HSPCs are at least partially protected by residual TAK1 signaling and/or a compensatory mechanism (such as cIAP). Consequently, we found that the mutant HSPCs are relatively sensitive to Smac mimetic (cIAP inhibitor) or TAK1 inhibitor treatment compared to healthy HSPCs. We verified these data in 5 *SF3B1* ^*mut*^ MDS patient samples [28]. Our study suggested that inhibition of cIAP or TAK1 signaling might be a useful strategy to selectively kill *SF3B1* ^*mut*^ HSPCs, especially when combined with other triggers for PANoptotic signaling such as an HMA or CBL0137. HMA and CBL0137 treatment might prime HSPCs to PANoptosis via reactivation of endogenous retroviruses [252, 253] and induction of mitochondrial DNA release [254, 255], respectively.

The major concern for Smac mimetic and TAK1 inhibitor treatments is to determine a dosage window that can selectively eliminate the disease clones with a tolerable effect on healthy HSPCs. In addition, cells dying from PANoptosis might cause systemic inflammatory syndrome, also known euphemistically as ‘cytokine storm’, as RIPK3‐mediated necroptosis is the major cause of systemic inflammatory syndrome. Fortunately, the dynamic PANoptosome makes the three types of PCD interconvertible, yet the inhibition of RIPK3 only prevents necroptosis while the cells will die of either apoptosis or pyroptosis when the PANoptosome is assembled. Thus, a RIPK3 inhibitor can be used to prevent systemic inflammatory syndrome in such cases.

## 5 Remarks on future studies of PANoptosis in MDS pathogenesis

Dysplasia, an inflammatory BM microenvironment and ineffective hematopoiesis are the three hallmark features of MDS. Increased PCD and impaired differentiation of BM HCs have been proposed as the causes of ineffective hematopoiesis. Consistent with this, our study suggested that PANoptosis contributes to ineffective hematopoiesis. It has also been suggested that ferroptosis and heme toxicity might also contribute to ineffective erythropoiesis, especially in MDS subtypes with ring sideroblasts, abnormal iron or heme accumulation in developing erythroblasts [256, 257]. Both PANoptosis and ferroptosis are inflammatory PCDs, which might explain the hyperinflammatory feature of MDS BM. Abnormal activation of Casp1 and 3 might impair the differentiation of erythroblasts by mediating the premature cleavage of GATA1 [258, 259]. In addition, RIPK1 and Casp8 in the mitotic ripoptosome regulate mitosis and chromosome stability, which might contribute to the dysplastic differentiation of HCs [185]. Future studies need to determine the direct association of PANoptosis and its associated signaling pathways with the three hallmark features of pathogenesis, disease risk states and treatment responses of MDS in genetically engineered mice with specific MDS mutations (such as *Sf3B1* ^*v700f/+*^, *Srsf2* ^*p95h/+*^ and *Tet2* ^*−/−*^) and/or more physiological *in vitro* preclinical MDS models such as human ‘ossicles’ for primary patient samples [260]. In addition, some of the details regarding PANoptotic functioning need to be further characterized by carefully taking the dynamic of PANoptosome formation and the kinetics of the three types of PCDs into consideration.

### 5.1 Is the PANoptosome formed in the same cell?

The concept of PANoptosis was originally discovered by detecting the co‐activation of the three PCD signaling pathways using Western blotting and interactions among the key components of the three PCD pathways using immunoprecipitation in tissue cells and was verified by observing maximal protection of cell death only when the key executors of all three PCD pathways (i.e., Casp1/11, Casp8, and Ripk3) were genetically or pharmacologically inactivated [33]. Due to the large numbers of cells used in the studies and the heterogenic features of these cells, there is a concern that the activation of the three PCD pathways might occur in different cells rather than in the same cell. Even if the activation of the three PCD pathways is detected in the same cell, still there is a possibility that the key components of the three pathways might not be located within the same complex. Unfortunately, there are only a few studies in which the formation of the PANoptosome was examined at a single cell level by determining the colocalization of the key compounds of the three PCD pathways (i.e., ASC, Ripk3 and Casp8) using immunofluorescence assays [261, 262]. In our study, we found the colocalization of ASC, p‐MLKL and active Casp3 in a few BM cells in almost all of the MDS samples we examined [28]. Future studies need to utilize higher resolution assays such as single‐cell imaging to capture the exact percentages of dying cells that have activation of one, two or all three PCD pathways. It also remains to be determined whether activation of one, two or all three PCD pathways represents the kinetic stages of PANoptosome formation or represents an individual type of PCD.

### 5.2 Is the PANoptosome formed simultaneously or sequentially?

The three PCDs have different kinetics. In TNFα‐ or LPS‐induced Casp8‐mediated apoptosis and Ripk3‐MLKL‐mediated necroptosis, RIPK1 oligomerization initiates RIPK3 recruitment via RHIM‐RHIM interactions and FADD/Casp8 recruitment via its DD domain, forming a ripoptosome [263, 264], triggering necroptosis within 2–4 h and apoptosis within 8–24 h [265]. In this context, both necroptotic and apoptotic signals can cause ASC‐NLRP3‐pyroptosis at later times by linking necroptosis and apoptosis with pyroptosis [266]. In noncanonical RIPK3‐initiated PANoptosis, the low‐polymerized RIPK3 oligomers rapidly recruit MLKL and assemble RIPK3‐MLKL to trigger necroptosis, whereas further progressively polymerizing RIPK3 homo‐aggregates facilitate the exposure of RIPK1‐DD to recruit FADD and assemble RIPK3‐MLKL‐RIPK1‐FADD‐Casp8 complexes [267]. In such a context, RIPK3‐initiated pyroptosis is dependent on Casp8‐mediated GSDMD cleavage independent of ASC‐Casp1 [53, 98, 100, 268]. LPS plus ATP is well‐known to stimulate canonical NLRP3 inflammasome‐pyroptosis. However, a longer time exposure of LPS plus ATP stimulation induces NLRP3‐driven PANoptosome formation [269]. In such a context, GSDMD‐induced pyroptosis often occurs rapidly, whereas MLKL activation is typically a much slower process [270]. This kinetic assembly of the PANoptosome together with the co‐existence of more than one PRR in the PANoptosome supports the two hits theory of PANoptosis [271]. In addition, in studying the *Nup98‐Hoxd13* transgenic mouse model, Wu et al. suggested that the heme metabolic, ferroptotic and senescence pathways were upregulated in the predisease and early disease stages, while the prominent roles of apoptosis, pyroptosis, and inflammasome signaling pathways were observed in the late stage [272]. Thus, a temporal dynamic analysis of pyroptotic, apoptotic, and necroptotic markers is needed at different disease stages for a better understanding of PANoptosis in MDS pathogenesis.

### 5.3 Does PANoptosis result from crosstalk among the PCD pathways?

Most PCD pathways are closely interconnected. For example, active MLKL triggers the NLRP3 inflammasome in a cell‐intrinsic manner [273, 274] and RIPK3 regulates the NLRP3 and pyrin inflammasome by its interaction with RIPK1 and CASP8, independent of its kinase activity [275, 276]. As is the case with Casp1, Casp8 also cleaves GSDMD and triggers pyroptotic cell death [100, 277]. Activation of the three types of PCD mediated by such crosstalk should be considered to be instances of PANoptosis as long as a PANoptosome is detected in the same cell because it is mediated by well recognized PANoptotic pathways. In addition, in Casp8‐deficient cells, only necroptotic and pyroptotic signals are detected; yet we still consider this to be PANoptosis because it is activated by canonical PANoptotic stimulators, and all major key components of the PANoptosome can be detected, with the exception of Casp8 [27]. However, in mitochondria‐mediated intrinsic apoptosis, active Casp3/7 promotes secondary necrosis by cleaving GSDME, similar to Casp1‐GSDMD‐mediated pyroptosis [278, 279, 280]. Such PCD cannot be considered to be PANoptosis because distinct mechanisms are involved in such PCD. For example, staurosporine is a well‐known inducer of intrinsic apoptosis through its inhibition of ATP‐competitive kinases, particularly the STE20 family of kinases, including those in the MST, GCK, and PAK subfamilies [281]. However, Sarker et al. showed that a higher dose of staurosporine also induces TNF‐dependent Ripk1‐Ripk3/Casp8‐mediated PANoptosis at later timepoints [282]. It needs to be determined whether such high‐dose staurosporine‐induced PANoptosis is dependent on mitochondrial apoptosis and its associated secondary necrosis or is activated by a completely novel mechanism.

### 5.4 In which types of cells does PANoptosis occur in MDS samples?

Due to technical limitations, most studies examined PCD in whole BM cells. Differentiation lineage and stage‐specific PCDs were only examined in *in vitro* culture and in a few animal models. Currently, we do not know in which lineage and differentiation stage of BM cells PANoptosis occurs in MDS patient samples. In addition, Casp3 is transiently activated in erythroid progenitors and early EBs, as well as maturing mast cells and megakaryocytes, while Casp8 is transiently activated in monocytes during the process of differentiation to macrophages [283]. Although such low levels of nonapoptotic activation of Casp3 or 8 are barely detected by the standard immunofluorescence assay, it needs to be taken into consideration in future analyses of PCD in BM cells.

## 6 Conclusions

PANoptosis has been better studied in several types of infectious diseases. Its roles in the pathogenesis of premalignant and malignant disorders are emerging. Our recent studies suggest that increased PANoptosis of BM HCs is one of the causes of ineffective hematopoiesis observed in MDS patients, and targeting PANoptosis might provide a novel treatment strategy for MDS. Thus, a better understanding of the signaling pathways that regulate PANoptosis and fully elucidating the molecular mechanisms by which BM HCs from MDS patients become hypersensitive to PANoptotic stimuli should help to develop more effective medications for this refractory disease.

## Conflict of interest

The authors declare no conflicting interests.

## Author contributions

RT, JL, CL, and JZ drafted the manuscript. PB and JZ helped to write and also edited and refined the manuscript.

## Supporting information


**Fig. S1.** A summary of two‐hits for PANoptosome assembly in different models.
**Table S1.** PANoptosis in infectious diseases.
**Table S2.** Studies demonstrate increased pyroptosis and necroptosis in MDS.
