# Supplementary material for: PANoptosis in the pathogenesis of myelodysplastic syndromes
Source: Mol Oncol. 2026 Jul 24:10.1002/1878-0261.70309. Online ahead of print. doi: 10.1002/1878-0261.70309 (PMC13396991; doi:10.1002/1878-0261.70309)
Supplement: Supplementary file 1 — Fig. S1. A summary of two‐hits for PANoptosome assembly in different models. Table S1. PANoptosis in infectious diseases. Table S2. Studies demonstrate increased pyroptosis and necroptosis in MDS. [file MOL2-9999-0-s001.docx]

**Supplementary information**

***Figure S1. A summary of two-hits for PANoptosome assembly in different models.***

**
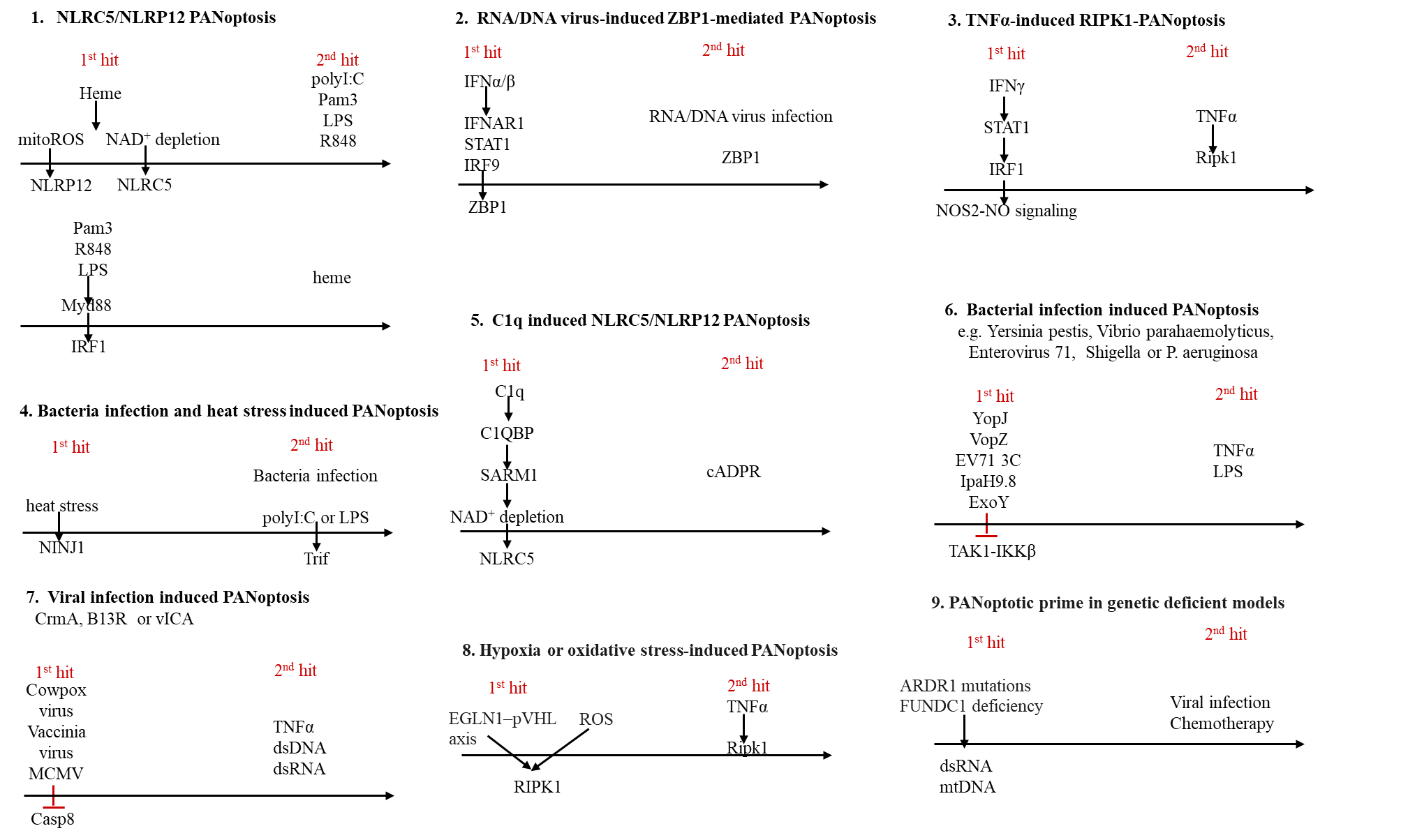
**

**Supplementary Table 1. PANoptosis in infectious diseases**

| **Pathogens** | **PANoptosome** | **PAMPs/DAMPs** | **Signaling and phenotypes** |
| --- | --- | --- | --- |
| Influenza virus (IAV) infection [[1-6](#_ENREF_1)]  Fungal pathogens  *C. albicans* *and A. fumigatus*[[7](#_ENREF_7)] | ZBP1-RIPK1/NLRP3/ASC/CASP8/CASP1/CASP6/RIPK3  The Zα1 and Zα2 domains of ZBP1 senses the ZRNAs and ZDNAs, and through its two RHIMs to interact with other RHIMs in RIPK1 and RIPK3. Casp6 is a key regulator of inflammasome activation. | Influenza vRNPs NP and PB1  Z-DNA and Z-RNA[[8](#_ENREF_8)]  IFNγ via IRF1 induces ZBP1 expression | RIPK3-MLKL necroptosis causes SIRS and contributes to death of the animal.  Apoptosis and pyroptosis are required for viral clearance, T- cell responses and pathogen infection control.  RIPK3 inhibitor UH15-38 treatment prevents mice from lethal dose of IAV. |
| Herpes simplex virus 1 (HSV1)[[9-12](#_ENREF_9)]  *F. novicida*[[12](#_ENREF_12)] | AIM2- ZBP1/Pyrin/ASC/CASP8/FADD/CASP1/RIPK3  AIM2 recognizes dsDNA via its C-terminal HIN domain. | Viral dsDNA  oHSV upregulates ZBP1 by inducing the accumulation of Z-RNA  Bacterium dsRNA | AIM2-dependent and NLRP3- and NLRC4-independent cleavage of Casp1.  ZBP1 cooperates with Pyrin drive AIM2-mediated Casp1 activation, cytokine release and cell death. |
| Coronavirus[[13-16](#_ENREF_13)]  Murine coronavirus Mouse hepatitis virus (MHV)[[15](#_ENREF_15)]  SARS-CoV[[17](#_ENREF_17)] | ZBP1-RIPK3-MLKL/CASP8/NLRP3  IFNγ and TNFα-  STAT1-IRF1-NOS2-NO  TLRs | z-RNA  Synergism of IFNγ and TNFα  LPS-TLR4  dsRNA-TLR3  ssRNA-TLR7  envelope protein-TLR2  ORF-3a and ORF-8b of SARS-CoV activates necroptosis  The binding of Vaccinia virus E3 protein dsRBD domain with viral dsRNA promotes the formation or stabilization of Z-RNA.[[18](#_ENREF_18)] | Inflammatory cytokine storm and PANoptosis forms a positive feedback loop which triggers inflammatory cell death, tissue damage, and mortality in SARS-CoV-2 infection and cytokine shock syndromes.  SARS pathology.  ORF8a and ORF9b trigger apoptosis; ORF7a activates NF-κB; ORF3b upregulates the expression of several cytokines and chemokines; ORF6 limits interferon production; ORF3a induces necrotic cell death; and ORF8b induces DNA synthesis and suppresses the expression of the viral envelope protein.[[17](#_ENREF_17), [19](#_ENREF_19)] ORF8b aggregates cause ER stress, mitochondrial dysfunction, and caspase-independent cell death.[[20](#_ENREF_20)] ORF8b interacts directly with and robustly activates the NLRP3 inflammasome.  MHV infection induces Ceacam1-dependent inflammatory cell death, PANoptosis, and cytokine release in BMDMs. |
| *Yersinia* [[21-24](#_ENREF_21)]  *Y enterocolitica, Y. pseudotuberculosis*, and *Y. pestis*  TAKi+TNFα[[25](#_ENREF_25)]  *Pseudomonas*, *Vibrio* and enteroviruses[[26-28](#_ENREF_26)] | RIPK1-FADD/CASP8/RIPK3/NLRP3/ASC/CASP1  NLRP12[[29](#_ENREF_29)] and TLR4 | YopJ inhibits TAK1, IKKβ, and MAPKK.  Bacterial nucleotidyl cyclase inhibits TAK1.  T3SS Effector inhibits TAK1  Enterovirus 71 3C cleaves TAK1/TAB1/TAB2 complex  TNFα  LPS-TLR4  Tetra-acylated lipid A | CASP8 induces cleavage of GSDMD to elicit pyroptosis and IL-1β release.  TLRs stimulate NLRP3-dependent activation of CASP1. |
| *Escherichia coli*, *Citrobacter rodentium*,  LPS in heat stress (HS) models[[30](#_ENREF_30)] | TRIF-ZBP1-RIPK1-FADD-CASP8-RIPK3-NCLP3-ASC-CASP1  NINJ1 in a major mediator of cell death | In response to HS, polyI:C or LPS induce robust cell death  Casp8 regulates NINJ1 oligomerization to drive PANoptosis in response to LPS plus HS | NINJ1 is a critical executioner in HS plus PAMP-induced PANoptosis and inflammatory cytokine releasing to drive pathology and mortality independent of other pore-forming executioner proteins, GSDMD, GSDME, and MLKL.  NINJ1 and CASP7 downstream of CASP8 and RIPK3. NINJ1 is critical for the rupture of the plasma membrane and release of endogenous molecules such as HMGB1 and LDH.[[31](#_ENREF_31), [32](#_ENREF_32)] Glycine treatment inhibited NINJ1 oligomerization. |
| *Salmonella enterica serovar Typhimurium*[[33](#_ENREF_33)]  *Listeria monocytogenes* [[33-35](#_ENREF_33)]  *P. aeruginosa* | NLRC4/NLRP3/ASC/Casp8/Casp1Casp11/Ripk1/Ripk3  NAIP5- or NLRC4 | T3SS effector proteins, such as AvrA, SspH1, SseL, GtgA, SpvC, SopB, and SseK1/4[[36](#_ENREF_36)]  LLO activates NLRP3 and compromises lysosomal | Bacterial clearance is reduced in mice lacking Casp1, -11, -12, and -8, and RIPK3 in response to systemic infection with a low dose of a growth-attenuated strain of Salmonella, resulting in mortality.[[34](#_ENREF_34)]  BMDMs deficient in NAIP5 or NLRC4 show compensatory activation of RIPK1 and MLKL in *P. aeruginosa* infected macrophages. |

**Supplementary Table 2. Studies demonstrated increased pyroptosis and necroptosis in MDS**

| **Type of PCD/assays** | **Participates** | **Conclusions** |
| --- | --- | --- |
| ***Increase of pyroptosis in MDS*** |  |  |
| Flow cytometry analysis of ASC specks,  co-localization of NLRP3 and a-Casp1,  western blotting analysis for a-Casp1 and ELISA for IL1β, IL18, S100A8/9.  Functional studies: Blocking S100A9 by a S100A9 high-affinity chimeric decoy receptor (CD33-IgG_1_) in S100A9Tg mice, *U2AF1*-*S34F* cells, and *Sf3b1-K700E* BM cells. [[37](#_ENREF_37)] | 10 MDS and 5 age-matched normal controls for mRNA protein analysis.  33 MDS and 12 controls for S100A9 analysis.  55 MDSs and 11 controls for HMGB1analysis. | MDS HSPCs overexpress inflammasome proteins and manifest activated NLRP3 inflammasome and Casp1.  Alarmin S100A9 is high in MDS patients which stimulates inflammasome formation, IL-1β/IL-18 generation and pyroptotic cell death by inducing TLR4- NADPH oxidase (NOX)-mediated ROS production, cation influx and β-catenin activation.  Knockdown of NLRP3 or Casp1, neutralization of S100A9, and pharmacologic inhibition of NLRP3 or NOX can all suppress pyroptosis, ROS generation, and nuclear β-catenin in MDSs and are sufficient to restore effective hematopoiesis. |
| confocal and electron microscopy to visualize, and flow cytometry to quantify plasma-derived ASC-specks in BM and PB plasm [[38](#_ENREF_38)] | Discovery cohort  177 MDS  29 healthy controls  Validation cohort 113 MDS  31 healthy controls | ASC-specks are released to BM and PB plasms upon pyroptotic lysis of BM cells which are directly correlated with S100-A8 and S100-A9 concentrations.  ASC-specks in PB plasm are significantly higher in PB and BM plasma of MDS patients versus healthy donors which can be serviced as a sensitive and specific candidate plasma biomarker of medullary pyroptosis and ineffective hematopoiesis in MDS patients. |
| ox-mtDNA[[39](#_ENREF_39)] | Discovery cohort  176 MDS  29 healthy controls  Validation cohort 113 MDS  30 healthy controls | ox-mtDNA is released from pyroptotic cells which can be readily quantified in patient plasma.  Levels of Ox-mtDNA in plasm are significantly increased in MDS patients than health controls and patients with other hematopoietic diseases.  Levels of ox-mtDNA are positively correlated with levels of S100A9, S100A8, and ASC-specks, which provide a sensitive index of medullary pyroptosis. |
| Functionally define the pathway:  ox-mtDNA-TLR9-MyD88-inflammasome-IFN-IRF7-ISG[[40](#_ENREF_40)] | 100 MDS PB  70 MDS BM | ox-mtDNA plays in hematopoietic potential by inducing the overexpression and engagement of TLR9.  Effectively neutralize extracellular ox-mtDNA-TLR9 signaling may suppress DNA-sensor-directed inflammation in the BM niche and possibly improve hematopoiesis. |
| ***Increase of necroptosis in MDS*** |  |  |
| Ripk1 and pMLKL staining and necroptotic morphology by electron microscopy.  *VavCreBaxBakBid* triple-knockout mice[[41](#_ENREF_41)] | 22 MDS | An inverse correlation between Ripk1 and Bid expression in several MDS.  Increase pMLKL+ necroptotic cells in BM samples of MDS patients.  Bid impacts necroptotic signaling through modulation of Casp8–mediated Ripk1 degradation.  *VavCreBaxBakBid* triple-knockout mice leads to unrestrained BM necroptosis and MDS driven by increased Ripk1.  Hematopoiesis and cytokine production in *VavCreBaxBakBid* triple-knockout mice can be restored by Ripk1 heterozygous knockout and TNFα blocking. |
| Immunofluorescence staining of BM core biopsies using cleaved caspase-3 and necroptosis markers RIPK1, pMLKL.[[42](#_ENREF_42)] | 28 MDS (15 low-grade)  29 controls | RIPK1 expression highly correlated with the distribution of CD71^+^ erythroid precursors but not with CD34^+^ blast cells.  High RIPK1 protein is correlated with downregulation of Casp8.  Increased expression of RIPK1 and pMLKL in low-grade MDS.  Necroptosis in the pathogenesis of MDS and as a potential biomarker for the diagnosis of low-grade MDS. |
| RNAseq BM CD34^+^ cells[[43](#_ENREF_43)] | 64 MDS or CMML | Expression levels of MLKL are upregulated in CMML and MDS.  Downregulation is associated with absence of response to HMAs.  Higher RIPK1 expression associated with shorter survival,  RIPK1/RIPK3/MLKL are potential therapeutic targets in MDS. |
| Casp8^-/-^ mice[[44](#_ENREF_44)] |  | Develop MDS-like disease due to elevated RIPK1 protein levels and necroptosis in BM cells. |
| *Ripk1*^HEM KO^ mice[[45](#_ENREF_45)] |  | Exhibit inflammation, HSPC loss, and BMF, which is partially ameliorated by a Ripk3 or Mlkl1 deficiency.  Ablation of IFNγ but not TNFα receptor signaling significantly extends survival of these mice. |
| qRT-PCR[[46](#_ENREF_46)] | 12 CHIP  47 LR-MDS and 14 HR-MDS | Low (14/17 *Sf3b1^mut^*) and high (8/8 del-5q) *IL1B* gene expression LR-MDS,  Majority of the inflammasome-related genes, including *IL1B*, were primarily expressed in the monocyte compartment; the highest levels of *IL18* expression were found in HSPCs.  Increased *IL18* expression in *SF3B1*-mutated MDS |
| *Tak1^KD^* mice[[47](#_ENREF_47)] | 25 MDS  5 healthy controls | Increase PANoptosis in bone marrow samples from MDS patients.  *Tak1^KD^* mice develop MDS like diseases which can be reversed by RIPK1 inhibition.  The differentiation defects of *SF3B1^mut^* erythroblasts from MDS patients can be reversed by RIPK1 inhibition.  SF3B1 mutant HSPCs from MDS patients are hypersensitive to TAK1 inhibitor treatment compared to health HSPCs. |

**References**

1. Kuriakose T, Man SM, Malireddi RK, Karki R, Kesavardhana S, Place DE, Neale G, Vogel P, Kanneganti TD: **ZBP1/DAI is an innate sensor of influenza virus triggering the NLRP3 inflammasome and programmed cell death pathways**. *Sci Immunol* 2016, **1**(2).

2. Nogusa S, Thapa RJ, Dillon CP, Liedmann S, Oguin TH, 3rd, Ingram JP, Rodriguez DA, Kosoff R, Sharma S, Sturm O *et al*: **RIPK3 Activates Parallel Pathways of MLKL-Driven Necroptosis and FADD-Mediated Apoptosis to Protect against Influenza A Virus**. *Cell Host Microbe* 2016, **20**(1):13–24.

3. Kesavardhana S, Kuriakose T, Guy CS, Samir P, Malireddi RKS, Mishra A, Kanneganti TD: **ZBP1/DAI ubiquitination and sensing of influenza vRNPs activate programmed cell death**. *The Journal of experimental medicine* 2017, **214**(8):2217–2229.

4. Zheng M, Karki R, Vogel P, Kanneganti TD: **Caspase-6 Is a Key Regulator of Innate Immunity, Inflammasome Activation, and Host Defense**. *Cell* 2020, **181**(3):674–687 e613.

5. Kesavardhana S, Malireddi RKS, Burton AR, Porter SN, Vogel P, Pruett-Miller SM, Kanneganti TD: **The Zalpha2 domain of ZBP1 is a molecular switch regulating influenza-induced PANoptosis and perinatal lethality during development**. *J Biol Chem* 2020, **295**(24):8325–8330.

6. Gautam A, Boyd DF, Nikhar S, Zhang T, Siokas I, Van de Velde LA, Gaevert J, Meliopoulos V, Thapa B, Rodriguez DA *et al*: **Necroptosis blockade prevents lung injury in severe influenza**. *Nature* 2024, **628**(8009):835–843.

7. Banoth B, Tuladhar S, Karki R, Sharma BR, Briard B, Kesavardhana S, Burton A, Kanneganti TD: **ZBP1 promotes fungi-induced inflammasome activation and pyroptosis, apoptosis, and necroptosis (PANoptosis)**. *J Biol Chem* 2020, **295**(52):18276–18283.

8. Zhang T, Yin C, Boyd DF, Quarato G, Ingram JP, Shubina M, Ragan KB, Ishizuka T, Crawford JC, Tummers B *et al*: **Influenza Virus Z-RNAs Induce ZBP1-Mediated Necroptosis**. *Cell* 2020, **180**(6):1115–1129 e1113.

9. Pham TH, Kwon KM, Kim YE, Kim KK, Ahn JH: **DNA sensing-independent inhibition of herpes simplex virus 1 replication by DAI/ZBP1**. *J Virol* 2013, **87**(6):3076–3086.

10. Guo H, Omoto S, Harris PA, Finger JN, Bertin J, Gough PJ, Kaiser WJ, Mocarski ES: **Herpes simplex virus suppresses necroptosis in human cells**. *Cell Host Microbe* 2015, **17**(2):243–251.

11. Hayes CK, Wilcox DR, Yang Y, Coleman GK, Brown MA, Longnecker R: **ASC-dependent inflammasomes contribute to immunopathology and mortality in herpes simplex encephalitis**. *PLoS Pathog* 2021, **17**(2):e1009285.

12. Lee S, Karki R, Wang Y, Nguyen LN, Kalathur RC, Kanneganti TD: **AIM2 forms a complex with pyrin and ZBP1 to drive PANoptosis and host defence**. *Nature* 2021, **597**(7876):415–419.

13. Karki R, Lee S, Mall R, Pandian N, Wang Y, Sharma BR, Malireddi RS, Yang D, Trifkovic S, Steele JA *et al*: **ZBP1-dependent inflammatory cell death, PANoptosis, and cytokine storm disrupt IFN therapeutic efficacy during coronavirus infection**. *Sci Immunol* 2022, **7**(74):eabo6294.

14. Karki R, Sharma BR, Tuladhar S, Williams EP, Zalduondo L, Samir P, Zheng M, Sundaram B, Banoth B, Malireddi RKS *et al*: **Synergism of TNF-alpha and IFN-gamma Triggers Inflammatory Cell Death, Tissue Damage, and Mortality in SARS-CoV-2 Infection and Cytokine Shock Syndromes**. *Cell* 2021, **184**(1):149–168 e117.

15. Zheng M, Williams EP, Malireddi RKS, Karki R, Banoth B, Burton A, Webby R, Channappanavar R, Jonsson CB, Kanneganti TD: **Impaired NLRP3 inflammasome activation/pyroptosis leads to robust inflammatory cell death via caspase-8/RIPK3 during coronavirus infection**. *J Biol Chem* 2020, **295**(41):14040–14052.

16. Li S, Zhang Y, Guan Z, Ye M, Li H, You M, Zhou Z, Zhang C, Zhang F, Lu B *et al*: **SARS-CoV-2 Z-RNA activates the ZBP1-RIPK3 pathway to promote virus-induced inflammatory responses**. *Cell Res* 2023, **33**(3):201–214.

17. Yue Y, Nabar NR, Shi CS, Kamenyeva O, Xiao X, Hwang IY, Wang M, Kehrl JH: **SARS-Coronavirus Open Reading Frame-3a drives multimodal necrotic cell death**. *Cell Death Dis* 2018, **9**(9):904.

18. Koehler H, Cotsmire S, Zhang T, Balachandran S, Upton JW, Langland J, Kalman D, Jacobs BL, Mocarski ES: **Vaccinia virus E3 prevents sensing of Z-RNA to block ZBP1-dependent necroptosis**. *Cell Host Microbe* 2021, **29**(8):1266–1276 e1265.

19. McBride R, Fielding BC: **The role of severe acute respiratory syndrome (SARS)-coronavirus accessory proteins in virus pathogenesis**. *Viruses* 2012, **4**(11):2902–2923.

20. Shi CS, Nabar NR, Huang NN, Kehrl JH: **SARS-Coronavirus Open Reading Frame-8b triggers intracellular stress pathways and activates NLRP3 inflammasomes**. *Cell Death Discov* 2019, **5**:101.

21. Orning P, Weng D, Starheim K, Ratner D, Best Z, Lee B, Brooks A, Xia S, Wu H, Kelliher MA *et al*: **Pathogen blockade of TAK1 triggers caspase-8-dependent cleavage of gasdermin D and cell death**. *Science* 2018, **362**(6418):1064–1069.

22. Sarhan J, Liu BC, Muendlein HI, Li P, Nilson R, Tang AY, Rongvaux A, Bunnell SC, Shao F, Green DR *et al*: **Caspase-8 induces cleavage of gasdermin D to elicit pyroptosis during Yersinia infection**. *Proc Natl Acad Sci U S A* 2018, **115**(46):E10888–E10897.

23. Malireddi RKS, Gurung P, Kesavardhana S, Samir P, Burton A, Mummareddy H, Vogel P, Pelletier S, Burgula S, Kanneganti TD: **Innate immune priming in the absence of TAK1 drives RIPK1 kinase activity-independent pyroptosis, apoptosis, necroptosis, and inflammatory disease**. *J Exp Med* 2020, **217**(3).

24. Malireddi RKS, Kesavardhana S, Karki R, Kancharana B, Burton AR, Kanneganti TD: **RIPK1 Distinctly Regulates Yersinia-Induced Inflammatory Cell Death, PANoptosis**. *Immunohorizons* 2020, **4**(12):789–796.

25. Malireddi RKS, Bynigeri RR, Mall R, Nadendla EK, Connelly JP, Pruett-Miller SM, Kanneganti TD: **Whole-genome CRISPR screen identifies RAVER1 as a key regulator of RIPK1-mediated inflammatory cell death, PANoptosis**. *iScience* 2023, **26**(6):106938.

26. He C, Zhou Y, Liu F, Liu H, Tan H, Jin S, Wu W, Ge B: **Bacterial Nucleotidyl Cyclase Inhibits the Host Innate Immune Response by Suppressing TAK1 Activation**. *Infect Immun* 2017, **85**(9).

27. Lei X, Han N, Xiao X, Jin Q, He B, Wang J: **Enterovirus 71 3C inhibits cytokine expression through cleavage of the TAK1/TAB1/TAB2/TAB3 complex**. *J Virol* 2014, **88**(17):9830–9841.

28. Zhou X, Gewurz BE, Ritchie JM, Takasaki K, Greenfeld H, Kieff E, Davis BM, Waldor MK: **A Vibrio parahaemolyticus T3SS effector mediates pathogenesis by independently enabling intestinal colonization and inhibiting TAK1 activation**. *Cell Rep* 2013, **3**(5):1690–1702.

29. Vladimer GI, Weng D, Paquette SW, Vanaja SK, Rathinam VA, Aune MH, Conlon JE, Burbage JJ, Proulx MK, Liu Q *et al*: **The NLRP12 inflammasome recognizes Yersinia pestis**. *Immunity* 2012, **37**(1):96–107.

30. Han JH, Karki R, Malireddi RKS, Mall R, Sarkar R, Sharma BR, Klein J, Berns H, Pisharath H, Pruett-Miller SM *et al*: **NINJ1 mediates inflammatory cell death, PANoptosis, and lethality during infection conditions and heat stress**. *Nat Commun* 2024, **15**(1):1739.

31. David L, Borges JP, Hollingsworth LR, Volchuk A, Jansen I, Garlick E, Steinberg BE, Wu H: **NINJ1 mediates plasma membrane rupture by cutting and releasing membrane disks**. *Cell* 2024, **187**(9):2224–2235 e2216.

32. Kayagaki N, Kornfeld OS, Lee BL, Stowe IB, O'Rourke K, Li Q, Sandoval W, Yan D, Kang J, Xu M *et al*: **NINJ1 mediates plasma membrane rupture during lytic cell death**. *Nature* 2021, **591**(7848):131–136.

33. Christgen S, Zheng M, Kesavardhana S, Karki R, Malireddi RKS, Banoth B, Place DE, Briard B, Sharma BR, Tuladhar S *et al*: **Identification of the PANoptosome: A Molecular Platform Triggering Pyroptosis, Apoptosis, and Necroptosis (PANoptosis)**. *Front Cell Infect Microbiol* 2020, **10**:237.

34. Doerflinger M, Deng Y, Whitney P, Salvamoser R, Engel S, Kueh AJ, Tai L, Bachem A, Gressier E, Geoghegan ND *et al*: **Flexible Usage and Interconnectivity of Diverse Cell Death Pathways Protect against Intracellular Infection**. *Immunity* 2020, **53**(3):533–547 e537.

35. Sundaram B, Karki R, Kanneganti TD: **NLRC4 Deficiency Leads to Enhanced Phosphorylation of MLKL and Necroptosis**. *Immunohorizons* 2022, **6**(3):243–252.

36. Shi C, Cao P, Wang Y, Zhang Q, Zhang D, Wang Y, Wang L, Gong Z: **PANoptosis: A Cell Death Characterized by Pyroptosis, Apoptosis, and Necroptosis**. *J Inflamm Res* 2023, **16**:1523–1532.

37. Basiorka AA, McGraw KL, Eksioglu EA, Chen X, Johnson J, Zhang L, Zhang Q, Irvine BA, Cluzeau T, Sallman DA *et al*: **The NLRP3 inflammasome functions as a driver of the myelodysplastic syndrome phenotype**. *Blood* 2016, **128**(25):2960–2975.

38. Basiorka AA, McGraw KL, Abbas-Aghababazadeh F, McLemore AF, Vincelette ND, Ward GA, Eksioglu EA, Sallman DA, Ali NA, Padron E *et al*: **Assessment of ASC specks as a putative biomarker of pyroptosis in myelodysplastic syndromes: an observational cohort study**. *Lancet Haematol* 2018, **5**(9):e393–e402.

39. Ward GA, McGraw KL, Abbas-Aghababazadeh F, Meyer BS, McLemore AF, Vincelette ND, Lam NB, Aldrich AL, Al Ali NH, Padron E *et al*: **Oxidized mitochondrial DNA released after inflammasome activation is a disease biomarker for myelodysplastic syndromes**. *Blood Adv* 2021, **5**(8):2216–2228.

40. Ward GA, Dalton RP, 3rd, Meyer BS, McLemore AF, Aldrich AL, Lam NB, Onimus AH, Vincelette ND, Trinh TL, Chen X *et al*: **Oxidized Mitochondrial DNA Engages TLR9 to Activate the NLRP3 Inflammasome in Myelodysplastic Syndromes**. *Int J Mol Sci* 2023, **24**(4).

41. Wagner PN, Shi Q, Salisbury-Ruf CT, Zou J, Savona MR, Fedoriw Y, Zinkel SS: **Increased Ripk1-mediated bone marrow necroptosis leads to myelodysplasia and bone marrow failure in mice**. *Blood* 2019, **133**(2):107–120.

42. Zou J, Shi Q, Chen H, Juskevicius R, Zinkel SS: **Programmed necroptosis is upregulated in low-grade myelodysplastic syndromes and may play a role in the pathogenesis**. *Exp Hematol* 2021, **103**:60–72 e65.

43. Montalban-Bravo G, Class CA, Ganan-Gomez I, Kanagal-Shamanna R, Sasaki K, Richard-Carpentier G, Naqvi K, Wei Y, Yang H, Soltysiak KA *et al*: **Transcriptomic analysis implicates necroptosis in disease progression and prognosis in myelodysplastic syndromes**. *Leukemia* 2020, **34**(3):872–881.

44. Liu S, Joshi K, Zhang L, Li W, Mack R, Runde A, Hagen PA, Barton K, Breslin P, Ji HL *et al*: **Caspase 8 deletion causes infection/inflammation-induced bone marrow failure and MDS-like disease in mice**. *Cell Death Dis* 2024, **15**(4):278.

45. Roderick-Richardson JE, Lim SE, Suzuki S, Ahmad MH, Selway J, Suleiman R, Karna K, Lehman J, O'Donnell J, Castilla LH *et al*: **ZBP1 activation triggers hematopoietic stem and progenitor cell death resulting in bone marrow failure in mice**. *Proc Natl Acad Sci U S A* 2024, **121**(4):e2309628121.

46. Schneider M, Rolfs C, Trumpp M, Winter S, Fischer L, Richter M, Menger V, Nenoff K, Grieb N, Metzeler KH *et al*: **Activation of distinct inflammatory pathways in subgroups of LR-MDS**. *Leukemia* 2023, **37**(8):1709–1718.

47. Zhang L, Li W, Thalla R, Ma R, Mack R, Kini AR, Runde A, Hagen PA, Barton K, Kosti-Schwartz J *et al*: **TGFbeta-activated kinase-1 knockdown in hematopoietic stem-progenitor cells causes PANoptosis and myelodysplastic syndrome-like disease in mice**. *Haematologica* 2026, **111**(4):1355–1367.
